# Spontaneous formation of autocatalytic sets with self-replicating inorganic metal oxide clusters

**DOI:** 10.1073/pnas.1921536117

**Published:** 2020-05-05

**Authors:** Haralampos N. Miras, Cole Mathis, Weimin Xuan, De-Liang Long, Robert Pow, Leroy Cronin

**Affiliations:** ^a^School of Chemistry, University of Glasgow, G12 8QQ Glasgow, United Kingdom

**Keywords:** autocatalysis, autocatalytic sets, self-assembly, molecular replication, origin of life

## Abstract

Here we show how a simple inorganic salt can spontaneously form autocatalytic sets of replicating inorganic molecules that work via molecular recognition based on the {PMo_12_} ≡ [PMo_12_O_40_]^3–^ Keggin ion, and {Mo_36_} ≡ [H_3_Mo_57_M_6_(NO)_6_O_183_(H_2_O)_18_]^22–^ cluster. These small clusters are able to catalyze their own formation via an autocatalytic network, which subsequently template the assembly of gigantic molybdenum-blue wheel {Mo_154_} ≡ [Mo_154_O_462_H_14_(H_2_O)_70_]^14–^, {Mo_132_} ≡ [Mo^VI^_72_Mo^V^_60_O_372_(CH_3_COO)_30_(H_2_O)_72_]^42–^ ball-shaped species containing 154 and 132 molybdenum atoms, and a {PMo_12_}⊂{Mo_124_Ce_4_} ≡ [H_16_Mo^VI^_100_Mo^V^_24_Ce_4_O_376_(H_2_O)_56_ (PMo^VI^_10_Mo^V^_2_O_40_)(C_6_H_12_N_2_O_4_S_2_)_4_]^5–^ nanostructure. Kinetic investigations revealed key traits of autocatalytic systems including molecular recognition and kinetic saturation. A stochastic model confirms the presence of an autocatalytic network involving molecular recognition and assembly processes, where the larger clusters are the only products stabilized by the cycle, isolated due to a critical transition in the network.

## Significance

Self-replication is an important property of life, yet no one knows how it arose, and the machinery found in modern cells is far too complex to have formed by chance. One suggestion is that simple networks may become able to cooperate and hence replicate together forming autocatalytic sets, but no simple systems have been found. Here we present an inorganic autocatalytic, based on molybdenum blue, that is formed spontaneously when a simple inorganic salt of sodium molybdate is reduced under acidic conditions. This study demonstrates how autocatalytic sets, based on simple inorganic salts, can lead to the spontaneous emergence of self-replicating systems and solves the mystery of how gigantic molecular nanostructures of molybdenum blue can form in the first place.

**Fig. 1.**
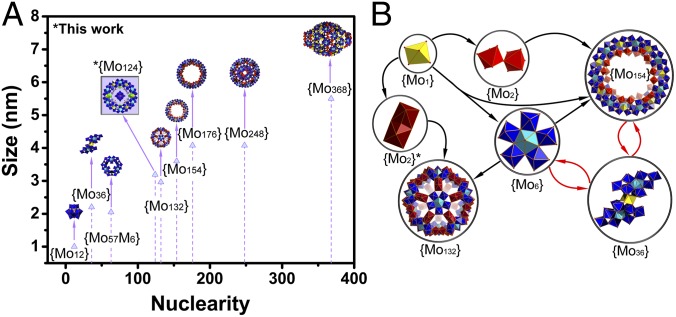
Size-nuclearity correlation and hypothesized embedded autocatalytic network. (*A*) Comparative representation of molybdenum-based family: [PMo_12_O_40_]^3–^, {PMo_12_}; [Mo^VI^_36_O_112_(H_2_O)_16_]^8–^, {Mo_36_}; [H_3_Mo_57_M_6_(NO)_6_O_183_(H_2_O)_18_]^22–^, {Mo_57_M}; [Mo^VI^_72_Mo^V^_60_O_372_ (CH_3_COO)_30_ (H_2_O)_72_]^42–^, {Mo_132_}; [Mo_154_O_462_H_14_(H_2_O)_70_]^14–^, {Mo_154_}; [H_16_Mo_248_O_720_(H_2_O)_128_]^16–^, {Mo_248_}; [H_16_Mo_368_O_1032_(H_2_O)_240_(SO_4_)_48_]^48–^, {Mo_368_}. The molybdenum-blue structure reported here, [H_16_Mo^VI^_100_Mo^V^_24_Ce_4_O_376_(H_2_O)_56_(PMo^VI^_10_Mo^V^_2_ O_40_)(C_6_H_12_N_2_O_4_S_2_)_4_]^5–^, {Mo_124_} or compound **1** is highlighted in the purple box. (*B*) Hypothesized embedded autocatalytic set which funnels mass from small {Mo_1_} (yellow polyhedra) monomers into corner-shared (light red), edge-shared (dark red) dimers {Mo_2_}, {Mo_6_} (blue/cyan polyhedra) building blocks, {Mo_36_} templates and {Mo_132_} Keplerate ball and {Mo_154_} molybdenum-blue wheel. The closed-loop nature of the autocatalytic sets enables the members of the set to be produced exponentially faster than competing products.

Biological self-replication is driven by complex machinery requiring large amounts of sequence information too complex to have formed spontaneously (1–4). One route for the emergence of self-replicators is via autocatalytic sets (5–8), which are collections of units that act cooperatively to replicate. Experimentally autocatalytic sets have been based on RNA, or peptides, and require sequence information, that is unlikely to emerge spontaneously itself (3, 4). Similarly, the design of directed molecular networks composed of molecules not used in biology gives insights into how complex self-organized systems build themselves (9), but again these systems are too complex to form randomly. The identification of examples of molecular-level organization of autocatalysis outside of biology, would give insights into how the universal "lifelike" chemistry can be (10), as well as allow the development of new approaches to the catalytic production and self-assembly of functional molecular nanomaterials (11, 12). Our previous investigations suggested that molybdenum blue chemical systems are governed by a spontaneously organized cooperative network of fast reactions. Specifically we had discovered an intermediate structure in which a central {Mo_36_} cluster appears to template the assembly of the surrounding {Mo_150_} ≡ [Mo^VI^_130_Mo^V^_20_O_442_(OH)_10_(H_2_O)_60_]^14–^ wheel (13). As a result we hypothesized that the formation of such complex gigantic inorganic clusters was only possible due to the utilization of a number of common building blocks (“Mo_1_,” “Mo_2_,” and “Mo_6_”) able to form embedded autocatalytic sets (Fig. 1).

This is because in general the formation mechanism of large clusters via the polymerization of molybdenum oxide cannot explain why only very specific products are formed since the utilized building-block library could in principle form thousands of structures of comparable stability via a combinatorial explosion process. Overcoming thermodynamic and geometry-imposed limitations requires the transfer of key molecular templating information during the formation cycle, leading to full conversion and finally production of very specific giant molecules. For example, a solution of sodium molybdate at pH 1.7 always produces a cluster containing 36 molybdenum atoms {Mo_36_} in the absence of any added ligands or reducing agents (14). When a reducing agent is used, it is possible to produce a family of reduced clusters where the nuclearity can increase up to a maximum of 368 and diverse structures such as nanosized spheres {Mo_132_}, wheels {Mo_154_}, capped wheels {Mo_248_} ≡ [H_16_Mo_248_O_720_(H_2_O)_128_]^16–^, and “lemon”-shaped {Mo_368_} ≡ [H_16_Mo_368_O_1032_(H_2_O)_240_(SO_4_)_48_]^48–^ clusters (Fig. 1). Even with a reducing agent less than 10 specific classes of large reduced molybdate clusters are known (Fig. 1) despite an incalculable number of possibilities, and the reactions that form the clusters are fast. Here we show that this system is an example of a spontaneously arising self-reproducing inorganic autocatalytic set, and that the autocatalytic nature of this process facilitates the formation of the well-known class of gigantic molybdenum-blue clusters. This is interesting because the formation mechanism of the class of inorganic gigantic protein sized molecules known as molybdenum blues has been a mystery, since the statistical formation of a plethora of clusters should prevent the formation of “magic numbers” of well-defined nanomolecules (15).

## Results and Discussion

**Fig. 2.**
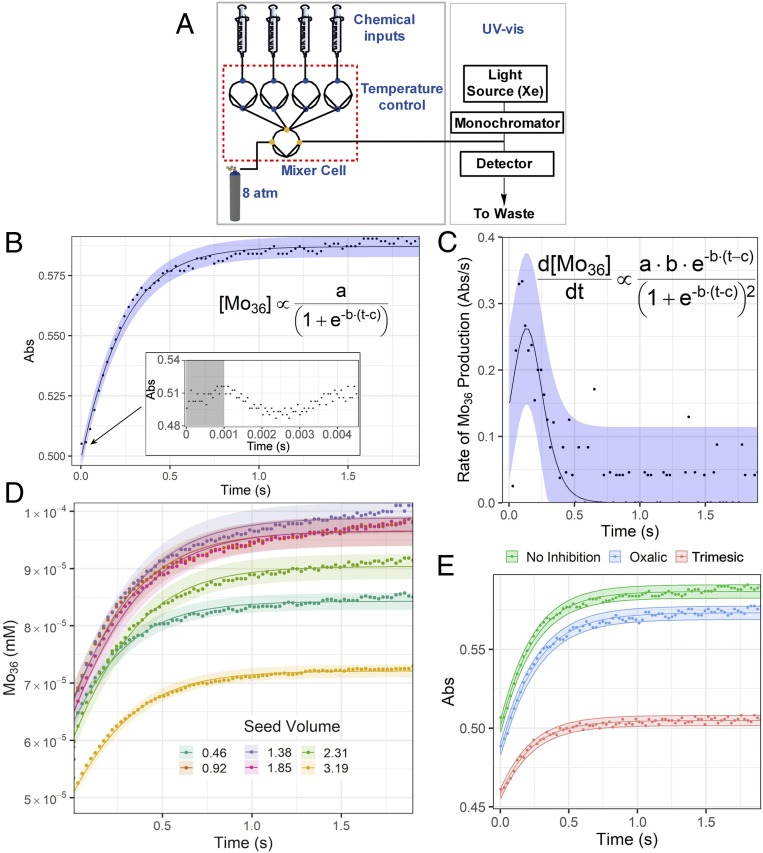
Experimental setup and spectroscopic data for {Mo_36_}. (*A*) Schematic of the stopped-flow spectrophotometer. The pumps are shown as circles connected to the valves. (*B*) Absorption vs. time profile of {Mo_36_} (in H_2_O at 24.3 °C), initial concentrations [Mo] = 0.25 M, [H^+^] = 0.047 M. (*Inset*) The lag time observed at the early stages of the reaction. (*C*) Rate of formation of {Mo_36_} as a function of time using a finite-difference method applied directly to the data. (*D*) Concentration vs. time profile of {Mo_36_} (in H_2_O at 24.3 °C), initial concentrations [Mo] = 0.25 M, [H^+^] = 0.047 M. The points represent the concentration profile vs. time of the same reaction mixture seeded with preformed {Mo_36_} 0.0016 M. (*E*) The molecular recognition is inhibited as a function of the number of the available hydrogen-bond sites provided by the inhibitors with two and three carboxylic acid groups, respectively.

To investigate the kinetics of the formation of the species we monitored the reactions using stopped flow with an ultraviolet (UV)-vis–based detection system (Fig. 2*A*). Here, the contents of syringes containing the reagents for cluster assembly are injected into an observation cell driven by a piston operating at 8 bar. Then the flow is stopped, and the reaction monitored. The first set of stopped-flow UV-vis data (*SI Appendix*, Figs. S1 and S2) was obtained from an acidified (pH ∼ 1.7) molybdate solution known to lead to the formation of the {Mo_36_} cluster. We set up the stopped-flow system so that it mixed freshly prepared solutions of Na_2_MoO_4_‧2H_2_O, (0.25 M) and aqueous HCl (0.047 M) in equal volumes, and the absorbance corresponding to the formation the {Mo_36_} cluster (350 nm) was monitored as a function of time. The increase of the concentration {Mo_36_} revealed a sigmoidal relationship (Fig. 2*B* and *SI Appendix*, Fig. S3), which is indicative of an autocatalytic process (Fig. 2*C*). We hypothesized that the autocatalysis of {Mo_36_} occurs via a molecular recognition process whereby the structure of one cluster acts as coordinate to other fragments allowing the structure to be formed via hydrogen-bonded and electrostatic interactions. To test this, we repeated the kinetic studies under the same conditions, but with 0.1 M of organic di- and triacids that could hydrogen bond to the cluster and inhibit the molecular recognition process (Fig. 2*D*). These experiments showed inhibition of the formation of {Mo_36_} when oxalic and trimesic acid were used. In this case the carboxylic acids, which are capable of binding to the constituents of the autocatalytic set in an unproductive manner, decrease the overall rate of the reaction.

An alternative way to interrupt the molecular recognition within the autocatalytic set is to prevent the formation or promote the destruction of the species responsible for the formation of the {Mo_36_} cluster. Thus, repetition of the stopped-flow experiment using Na_2_MoO_4_‧2H_2_O, (0.25 M) and diluted HCl (0.001 M, decreased from 0.047 M), resulted in a reaction mixture with pH value > 4.5 where the formation of {Mo_36_} was slowed dramatically (*SI Appendix*, Fig. S4). Additionally, when we reduced solution of Na_2_MoO_4_‧2H_2_O, (0.075 M) and HCl (0.1 M) with 0.023 M of Na_2_S_2_O_4_ (this is enough reducing agent to ensure that >60% of the molybdenum centers are reduced from 6+ to 5+) the fast formation of the {Mo_36_} was observed followed by rapid decomposition in under 1 s (*SI Appendix*, Fig. S5). These observations demonstrate the dependence on molecular recognition (15) in driving the rates observed initially during the formation of the {Mo_36_} cluster.

A key feature of self-replicating and autocatalytic sets is that during the initial stages of the reaction, the process occurs primarily via an uncatalyzed pathway. However, once the product in solution reaches a critical concentration, then the autocatalytic cycle begins to operate. Therefore, the presence of presynthesized {Mo_36_}, at the beginning of the reaction (*t* = 0), should result in no kinetic lag/induction period in the rate profile for the reaction, and an increase of the initial rate. In order to demonstrate this effect, Na_2_MoO_4_‧2H_2_O and HCl were reacted under identical conditions described previously, but with the addition of 1–3 mL of preformed {Mo_36_} 1.6 × 10^−3^ M (see *SI Appendix*, section 3–3 for details and *SI Appendix*, Fig. S6). As shown in *SI Appendix*, Figs. S7 and S8, autocatalyst saturation occurs after addition of 3 mL of preformed {Mo_36_} (1.6 × 10^−3^ M), inducing maximization of the self-propagated rate of {Mo_36_}, as is expected (16).

**Fig. 3.**
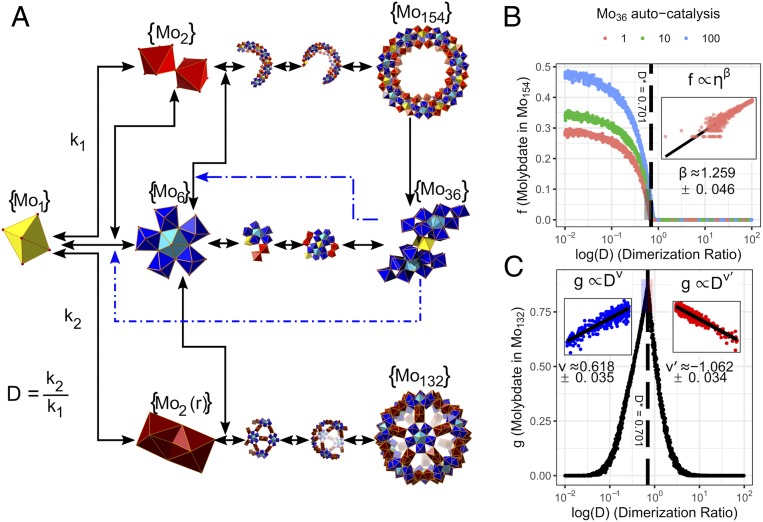
Scheme of stochastic model. (*A*) Wiring diagram representation of the stochastic kinetic model, showing the most relevant steps in the reaction network. The black arrows represent mass flow while the blue dotted arrows represent the effect of templates. (*B*) The model exhibits a critical transition to the formation of the giant nanostructure {Mo_154_}, the scaling relation near the critical point is shown in the *Inset*. (*C*) Near the critical point the formation of the other giant nanostructure {Mo_132_} is maximized, showing critical scaling near the transition, with different scaling coefficients on either side of the transition. Simulation parameters for this figure are shown in *SI Appendix*, Table S1; the results shown here are the time-average values for 3,000 simulations.

In order to explore the mechanism of self-assembly of the clusters, and to understand why only a finite number of very complex products are observed, we developed a stochastic model to simulate the formation of the clusters using a kinetic Monte Carlo approach, based on mass action (17). In principle this modeling approach requires estimates of the reaction rate constants for every possible reaction between molecular intermediates and efficient computational methods do not currently exist to extract the reaction rate constants from the time series of the molecular abundances, particularly given the large number of reactions. To avoid this issue, we aimed to model the dynamics phenomenologically by deriving a minimal set if assumptions about the underlying kinetics from the observed empirical properties of the system. To do this we modeled every possible reaction between the monomers and intermediates as reversible reactions. This model only considered the structure and the nuclearity of the molecules but not the exact composition (e.g., two different intermediates with the same number of Mo atoms but different composition are represented as the same intermediate), the only exception to this is that we model two different molybdenum dimer molecules, the corner-bonded Mo_2_ dimer, and the reduced, edge-bonded Mo_2_(r) dimer, which play different structural roles and appear in the Mo_154_ and Mo_132_, respectively. In the model, each reaction is reduced to its constituent steps such that only unimolecular reactions (in the case of degradation, A → B + C) and bimolecular (in the case of synthesis, A + B → C) are used, meaning that all implemented reactions are either first or second order. The reaction rates were determined by the associated reaction rate constants, as well as the concentration of reactants, and in the case of bimolecular reactions the reduced mass of the reactants, temperature, and volume of solution; schematic representation of this process is shown in Fig. 3*A*. All unimolecular reactions have an associated rate constant of 1.0 unless otherwise stated. The parameter D controls the ratio between the rate constant associated with formation of the corner-bonded dimer to the rate constant for the formation of the edge-bonded (reduced) dimer. Previous experiments have shown that large {Mo_132_} and {Mo_154_} clusters are stable to degradation (18). To model this stability we assigned the rate constants associated with degradation of those molecules, k_d_ << 1.0. To assign the bimolecular reaction rate constants we used a progression of different schemes which relied on the experimental observations.

The first scheme involved setting every bimolecular rate constant to the same value and ignoring any effects from templating. Under this scheme the {Mo_36_} structure formed, albeit in relatively low abundance, and the larger clusters did not form, even if the bimolecular rate constants were set to a relatively high value, owing to the combinatorically large number of possible products. We next included the effect of templating, by assuming that bimolecular reactions between intermediates bound to a template proceeded 10.0× faster than reactions without a template. We included this effect to account for the formation of {Mo_154_} (templated by {Mo_36_}), which was described in previous studies (13), and for the formation of {Mo_6_}, which was proposed as a mechanism responsible for the autocatalytic properties of the {Mo_36_} structure described above. While we found that including the effect did not ensure the formation of {Mo_154_}, the intermediate compounds between the {Mo_36_} and the {Mo_154_} or {Mo_132_} formed readily, those intermediates degraded before forming complete structures. This resulted in a combinatorial explosion in which many nanostructures nucleate but dissociate completing. This limitation cannot be easily overcome by increasing the stability of all intermediates, which only serves to preferentially increase the abundance of low nuclearity clusters, trapping building blocks “downstream” (*SI Appendix*, Figs. S26 and S27, which show how increasing the forward reaction rate actually reduces the abundance of higher nuclearity clusters).

**Fig. 4.**
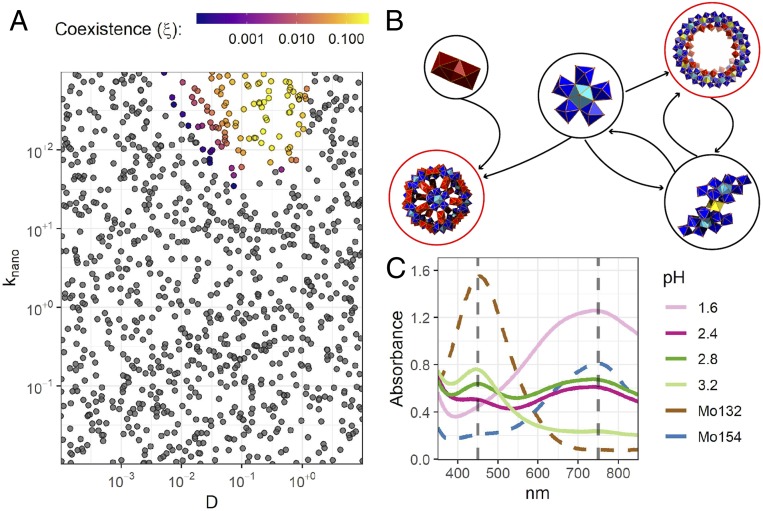
Stochastic model predictions. (*A*) The model predicts that the {Mo_154_} and {Mo1_32_} nanostructures can coexist for a narrow range of parameters. The time-average coexistence value is shown for 1,000 simulations with different values. This result was validated using UV-vis spectroscopy. (*B*) Assembly of the {Mo_132_} Keplerate ball from the {Mo_2_} with the {Mo_6_} and the formation of the {Mo_154_} wheel via the {Mo_6_} which also forms the {Mo_36_} wheel template. (*C*) The spectra presented in dashed lines were obtained using crystals of preformed {Mo_132_} Keplerate ball (brown line) and {Mo_154_} (blue line), respectively. Simulations parameters are shown *SI Appendix*, Table S1.

To overcome the frustrated formation of larger structures without fine-tuning the model, an additional free parameter, k_nano,_ was included. We assumed that the rate constant for bimolecular reactions between intermediates increased for reaction in which product molecules have few free-bonding sites. In this model k_nano_ measures the relative increase due this effect, such that when k_nano_ is higher products with fewer free-bonding sites are preferred over those with more free-bonding sites, while when k_nano_ is lower, the number of free-bonding sites does not bias the product formation. This could, for example, represent the fact that a nearly complete structure serves to coordinate building blocks, pulling them into gaps in the structure. By including this parameter, the model reproduces the phenomenological features of the physical system with a large range of parameters, without fine-tuning this large range of parameters individually, for example, the observation of the formation of {Mo_154_} and the autocatalytic nature of the {Mo_36_} template. In typical simulations the abundance of {Mo_154_} remains zero for a time followed by a brief period of exponential growth due and subsequent saturation (*SI Appendix*, Fig. S32). This feature is also seen in experimental data when the solution is not seeded with {Mo_36_}. To validate these assumptions, we investigated a hypothesis suggested by this model which could be readily measured in the laboratory. Specifically, the model predicts the coexistence of {Mo_132_} and {Mo_154_} for a narrow value of the dimerization ratio (D). In the experimental system D is controlled by the pH of the solution. Indeed, by careful investigation of the variation of the pH of the system, we were able to observe a transition between the two nanostructures including values at which they coexisted, thereby validating our model (Fig. 4).

Our model also allowed us to explore the dynamical consequences of different assumptions surrounding the formation of large nanostructure structures. For example, it was proposed that {Mo_36_} templates the formation {Mo_6_} building blocks, providing a clear mechanism for the autocatalytic behavior observed for the {Mo_36_} structure. However, due to the timescales associated with the formation it is difficult to test this assumption experimentally, but we can explore the consequences of including or excluding this dynamical feature using our model. We found that without the embedded autocatalytic cycle, the abundance of the {Mo_36_} was vanishingly small (*SI Appendix*, Fig. S28). Increasing the catalytic effect of the {Mo_36_} on the formation of {Mo_6_} resulted in higher steady-state abundance for the {Mo_154_} wheel. Importantly, we find that the formation of both the {Mo_154_} and {Mo_132_} structures are sensitive to the relative rate of dimerization, which is known to be controlled by the reducing environment and pH of the solution. For more oxidized solutions the (lower relative dimerization rates) the {Mo_154_} wheel forms in high yields until a critical threshold above which the wheel cannot form due to a lack of corner-shared {Mo_2_} dimers. Thus, there is a critical transition between two different macroscopic states of the system, one in which the wheel forms and maintains a steady-state abundance and one where the mean abundance of the {Mo_154_} wheel is zero. This transition is driven by the autocatalytic nature of the {Mo_36_} template and exhibits critical scaling near the transition (shown in Fig. 3 *B* and *C*, *Insets*) The critical scaling exponents do not appear to be rational numbers nor do they correspond to any known universality class. As the rate of reduced dimer formation increases above this threshold, the net yield of the {Mo_132_} in simulations increases until the formation of {Mo_6_} is affected, at which point the yield falls quickly to zero. This phenomenon is observed qualitatively in physical experiments by the progressive reduction of the solution (18).

Using the result of our model we can now propose a general mechanism underlying the assembly of highly specific giant structures from otherwise unconstrained monomers. The formation of {Mo_36_} has a downstream effect by catalyzing the formation of {Mo_6_} units and thereby catalyzing the formation of itself; however, it also has an upstream effect of stabilizing the formation of {Mo_154_} rings. By catalyzing the formation of {Mo_6_}, the {Mo_36_} catalysts ensure both its own autocatalytic formation and an abundance of the {Mo_6_} units generating the necessary conditions for the robust formation of {Mo_154_}. The combination of these two catalytic effects in a single molecule results in the truncation of an otherwise combinatorically large product space into the highly specific set of compounds, and this general phenomenon is responsible for the formation of the family of gigantic reduced molybdenum–oxide clusters.

To test this idea, we explored the kinetics of formation of the oldest known metal–oxo cluster, the Keggin ion, using stopped flow in an identical manner as for {Mo_36_}. As before the first sample container was loaded in this case with freshly prepared solutions of Na_2_MoO_4_‧2H_2_O, (1.4 M), but in addition the phosphate heteroanion, vital for the formation of the Keggin, was included (0.06 M of H_3_PO_4_) while the second container was loaded with HClO_4_ (4 M) which were mixed in equal volumes. Then the formation of the {PMo_12_}-Keggin cluster was monitored as a function of the time at 440 nm. Remarkably, a sigmoidal trend was also observed, indicating autocatalysis (*SI Appendix*, Fig. S17). To further verify the observation of autocatalysis, we repeated the above experiment in the absence of H_3_PO_4_ or in the presence of H_2_SO_4_ (0.06 M) since it is known that PO_4_^3–^ is the most effective template for the formation of the Keggin species (*SI Appendix*, Fig. S18) (19). This modification had a detrimental effect on the formation of {PMo_12_} species. Finally, seeding experiments with preformed 0.01 M {PMo_12_} eliminated the induction period and increased the amount of the final product produced within the same timescale. Kinetic saturation was observed where the initial rate of the {PMo_12_} kept increasing up to the addition of 1 mL of preformed catalyst before a plateau was reached, followed by a dramatic decrease (*SI Appendix*, Figs. S19 and S20).

**Fig. 5.**
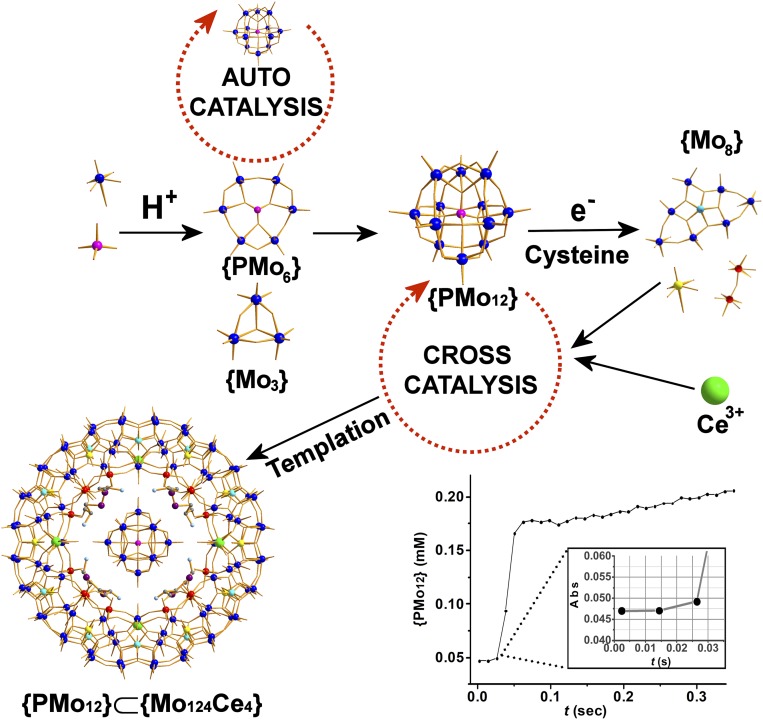
Stochastic model predictions. Scheme of auto/cross-catalytic sequence for **1**. Scheme showing the templated assembly of the {PMo_12_}⊂{Mo_124_Ce_4_} MB nanoring. Color scheme: {Mo_1_}, yellow; {Mo_2_}, red; {Mo_8_}, blue (central MoO_7_ pentagonal bipyramid, cyan); P, pink. The building blocks used in the formation of the Keggin species are postulated as {Mo_3_} and {PMo_6_}. Absorption vs. time profile of {PMo_12_} (in H_2_O at 24.3 °C), initial concentrations [Mo] = 0.7 M, [H^+^] = 0.06 M. The circles are experimental data points. (*Inset*) The lag time observed at the early stages of the reaction.

Given the ability of the {Mo_36_} to catalyze its own formation, as well as cross-catalytically template the {Mo_154_} molybdenum blue, we wondered if the {PMo_12_} Keggin could also be used to template a new type of Mo blue. By adding the {PMo_12_} to a reduced acidified solution of Ce_2_O_3_·7MoO_3_·6H_2_O we were able to quickly isolate and crystallize a ring with 124 Mo atoms. The ring was crystallized complete with the {PMo_12_} Keggin template and can be formulated as: (C_6_H_14_N_2_O_4_S_2_)_4_K[H_16_Mo^VI^_100_Mo^V^_24_Ce_4_O_376_(H_2_O)_56_(PMo^VI^_10_Mo^V^_2_O_40_)(C_6_H_12_N_2_O_4_S_2_)_4_]·200H_2_O {PMo_12_} ⊂ {Mo_124_Ce_4_} **1**, molybdenum-blue wheel (*SI Appendix*, section 4–1) (Fig. 5). Single-crystal X-ray structural analysis reveals that **1** crystalizes in the space group *C*2/*m* and features a nanoring {Mo_124_Ce_4_} (20), composed of 12 {Mo_8_} units, 8 {Mo_2_} units, 12 {Mo_1_} units, 4 {Ce(H_2_O)_5_} units, and 4 cystine molecules, with a {PMo_12_} Keggin cluster trapped in the center (*SI Appendix*, Fig. S13). The four Ce^3+^ ions are distributed symmetrically on the two ends of both the upper and lower rims of the {Mo_124_Ce_4_} cluster, such that the *C*_2_ symmetric ring has an oval-shaped opening with outer and inner ring diameter of about 29 and 19 Å, respectively. The {PMo_12_} Keggin cluster resides in the middle of the ring on a *C*_2_ axis and is anchored in place by a number of N−H···O hydrogen bonds formed with the 4 coordinated cystine ligands grafted onto the inner ring of {Mo_124_Ce_4_}.

Bond valence sum calculations were carried out on all of the Mo and O centers, revealing that **1** is composed of a 24-electron reduced anionic ring containing 16 singly and 56 doubly protonated oxygen atoms and that the {PMo_12_} host is 2-electron reduced. Crucially, we determined that the guest ⊂ host supramolecular complex forms unexpectedly fast and we could detect formation of single crystals 20 min after the completion of the synthetic procedure discussed in *Materials and Methods* below (*SI Appendix*, Figs. S14 and S15). Additionally, the synthesis in the absence of the {PMo_12_} did not give rise to the formation of the {Mo_124_Ce_4_} ring, suggesting that Keggin is also able to cross-catalyze the formation of the molybdenum-blue ring via a templation effect (Fig. 5 and *SI Appendix*, section 4–1), in a similar manner to the {Mo_36_} cluster in the case of the {Mo_154_} molybdenum-blue family. Addition of different concentrations of the Keggin ion template were also found to promote the formation of larger quantities of compound **1**, consistent with the catalytic template effect (*SI Appendix*, Fig. S35). The rapid self-assembly of the distinct {Mo_36_} and {PMo_12_} clusters can be explained by an autocatalytic self-replicating process requiring molecular recognition; additionally, when the same experiments were carried out in deuterated solvents the formation rate was slowed down, reflecting weaker hydrogen-bonded interactions (*SI Appendix*, Figs. S22–S24).

## Conclusions

The results presented here show that the formation of an autocatalytic set which embeds molecular template transfer processes can form with a simple inorganic system. We demonstrate that the autocatalytic formation of {Mo_154_} rings exhibits a critical transition in response to the reduction potential and pH of the solution, due to the coupling between the molecular autocatalyst {Mo_36_} and the formation of {Mo_154_}. The very fast kinetics associated with the molybdate system allows replicators to emerge whereas the tungstate system produces many more products, especially upon reduction, further confirming our hypothesis (*SI Appendix*, Fig. S34). Thus, we hypothesize that the formation of molybdenum nanostructures represents a unique class of self-organized criticality (21). All previous autocatalytic sets known are derived from known biology but this study shows how autocatalytic sets, based on simple inorganic salts, can spontaneously emerge which are capable of collective self-reproduction outside of biology (22). Finally, the unveiled knowledge and processes discovered in this inorganic system can be extrapolated and used constructively for the discovery of forms of materials and processes such as templated faceting of nanoparticles which can be finely controlled at the molecular level with direct consequences for their final properties or even emergence of new ones.

## Materials and Methods

All chemical reagents and solvents were purchased from Sigma-Aldrich Chemicals and used without further purification. All of the solutions were freshly prepared and used within 2 h.

The reported stopped-flow UV-vis spectroscopic experiments were conducted using an SX20 stopped-flow spectrophotometer (Applied Photophysics). Single-crystal diffraction data were collected at 150(2) K using a Bruker AXS Apex II [λ(MoKα) = 0.71073 Å] equipped with a graphite monochromator. The simulations in our stochastic kinetic model were developed using an implementation of the Gillespie algorithm written in Julia (v1.0 later) with the packages, dataframes, CSV, combinatorics, JSON, and Random and R (V3.5 or later for analysis) with the packages ggplot2, dplyr, and tidyr. The analysis was done using R via a Jupyter notebook and the full code and data for the simulations are available. More experimental details can be found in *SI Appendix*.

### Data Availability Statement.

All relevant data present in this publication can be accessed at https://github.com/croningp/clusterautocatalyticsets. Crystallographic data for compound **1** (CCDC 1916468) can be obtained free of charge from the Cambridge Crystallographic Data Centre, 12, Union Road, Cambridge CB2 1EZ; deposit@ccdc.cam.ac.uk.

## Supplementary Material

Supplementary File

Supplementary File
