# Supplementary material for: Spontaneous formation of autocatalytic sets with self-replicating inorganic metal oxide clusters
Source: Proc Natl Acad Sci U S A. 2020 May 5;117(20):10699–705. doi: 10.1073/pnas.1921536117 (PMC7245103; doi:10.1073/pnas.1921536117)
Supplement: Supplementary File [file pnas.1921536117.sapp.pdf]

Supplementary Information for

Spontaneous formation of autocatalytic sets with self-replicating inorganic metal oxide clusters

Haralampos N. Miras, Cole Mathis, Weimin Xuan, De-Liang-Long, Robert Pow and Leroy Cronin

School of Chemistry, University of Glasgow, Joseph Black Building, University Avenue, Glasgow G12 8QQ, U.K.

*Leroy Cronin

Email: Lee.Cronin@glasgow.ac.uk

This PDF file includes:

Supplementary text
Figures S1 to S35
Tables S1 to S3

www.pnas.org/cgi/doi/10.1073/pnas.1921536117

1. General Materials and Methods

All chemical reagents and solvents were purchased from Sigma-Aldrich Chemicals and used without further purification. All the solutions were freshly prepared and used within two hours.

The reported experiments were conducted using an SX20 stopped flow spectrophotometer (Applied Photophysics). The setup consists of a light source (150 W Xe lamp), programmable monochromator (enabling acquisition of time-resolved absorbance spectra and steady-state spectral acquisition), optimised detector for absorbance kinetics, temperature control chamber, mixing chamber and 20 μL volume cell (dead-time: 1 ms), as shown in Supplementary Figures 1 and 2. The experimental data were collected and processed using the ProData software supplied by Applied Photophysics.

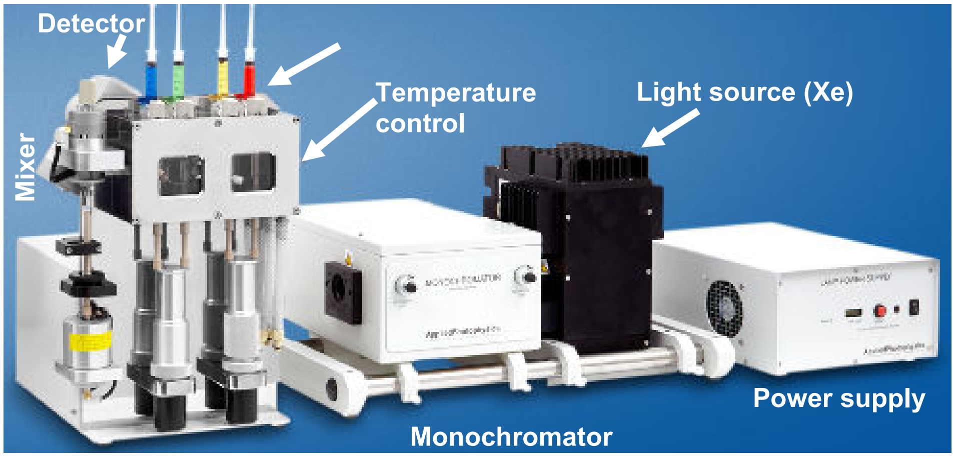

Figure S1. Image of the stopped flow system used for our experiments. The stock solutions and the relevant concentrations were: (a) $Na_2MoO_4·2H_2O$, [Mo] = 0.5 M; (b) HCl, $[H^+]$ = 0.1 M; (c) $Na_2S_2O_4$, $[e^-]$ = 0.04 M; (d) oxalic acid, [ox] = 0.18 M; (e) 1,3,5-$C_6H_3(COOH)_3$, [Tri] = 0.18 M.

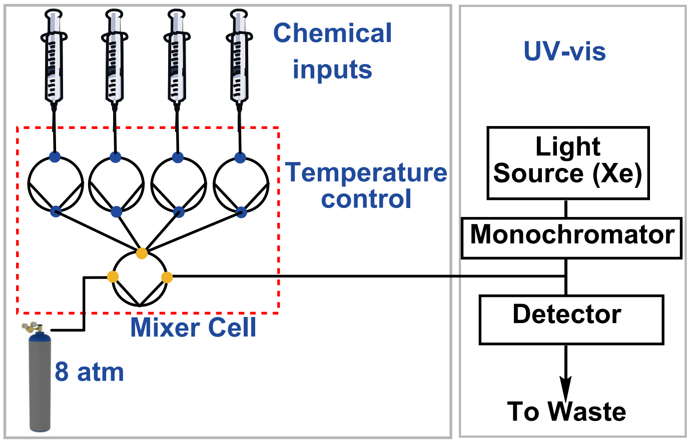

Figure S2. Schematic representation of the Stopped-flow spectrophotometer.

The chemical inputs have been used to supply the freshly prepared solution of the starting materials necessary for the investigation of the reaction of interest. The starting materials were loaded in the temperature controlled (23.4 ^{o}C) compartments which left to equilibrate for 5 minutes. Then under a pressure of 8 bar the reagents were mixed and promoted to the cell where the UV-vis spectrum was recorded at pre-defined time scales.

2. Definition and signs of autocatalysis

Autocatalysis is a general term used to describe an experimentally observable phenomenon in homogeneous chemical systems where a product of the chemical system catalyses the same reaction. Additionally, self-replication phenomena might take place at the same time, in the case where the product or species of the autocatalytic set template their own formation which results ultimately in the amplification of the reaction rate. More specifically, a marked increase of the reaction rate takes place as a function of time followed by a considerable decrease upon formation of considerable amount of product. The use of the term is appropriate only for chemical systems considered under constant temperature and pressure. Thus, highly exothermic reactions (eg. explosions) are excluded since in this case the observed rate increase is due to the rapid temperature rise. To identify an autocatalytic chemical system a series of signature are required inlcuding:

1. Exponential (sigmoidal) product vs. time curve with induction period.
2. Inhibition of molecular recognition and consequent deceleration of the reaction.
3. Rate increase and elimination of induction period upon seeding of the reaction mixture with pre-formed product.
4. Kinetic saturation of the system and deceleration exceeding a specific ratio limit.

In this work we demonstrate for the first time the existence of autocatalytic sets in all-inorganic systems and discuss their crucial role for the formation of nanosised Molybdenum Blue (MB) species.

3: Investigation of autocatalytic phenomena in MB chemistry.

Based on our previous experimental observations that the formation of the molybdenum blue species is controlled by a seemingly more complicated set of reactions rather than an oleosis/oxoleosis and reduction driven assembly process. More specifically, it was shown that the formation of the MB wheels is templated by the smaller $\{Mo_{36}\}$ cluster formed prior to the reduction and further assembly of the system. Since the overall assembly process masks a complex network of underlying reactions, we tried to isolate and investigate individually the fast processes that take place. Initially, we investigated the formation the $\{Mo_{36}\}$ because it is responsible for the templated synthesis of the nanosised MB wheels. Using the definition of autocatalysis/replication, as stated in SI-2, we explored the autocatalytic nature of the $\{Mo_{36}\}$ structure by confirming each of the four experimental signatures.

3-1: Concentration vs. time profile of the $\{Mo_{36}\}$ cluster formation

The first interesting observation in relation to the formation of the $\{Mo_{36}\}$ cluster was provided by the exponential growth (Supplementary Figure 3a) and subsequent kinetic saturation (see SI-3-4) of $\{Mo_{36}\}$ using time series data. The experimental data were collected and processed using the ProData software supplied by Applied Photophysics. The sample containers were loaded with freshly prepared solutions of $Na_2MoO_4 \cdot 2H_2O$, (0.25 M) and HCl (0.047 M) and were mixed in equal volumes and the absorbance corresponding to the formation the $\{Mo_{36}\}$ cluster (350 nm) was monitored as a function of the time. The overall scan time was set to t=2 sec.

A nonlinear regression algorithm was used to fit the absorption data to a sigmoidal curve of the form: $[Mo_{36}] = [Mo_{36}]_0 + a/(1+\exp(-b(t-c)))$, where a, b and c are fit parameters. A critical signature of autocatalysis is the rapid increase in reaction rate followed by the subsequent saturation and decrease in rate. To check for this effect in the data, we used the same regression method to fit the first derivative of the absorption measurements, effectively fitting the rate of the reaction. The derivative of the experimental data was calculated using a finite difference method and plotted in Supplementary Figure 3b. This set of data was fit to the first derivative of the sigmoid function used to fit the adsorption data,

$$\frac{d[Mo_{36}]}{dt} = \frac{a * b * \exp(-b(t-c))}{\left(1+\exp(-b(t-c))\right)^2}. \qquad (1)$$

Given the paramount importance of confirming this sigmoidal behaviour, and the inherent uncertainty in fitting data to a first derivative, the evidence of this fit needs to be compared against plausible

alternatives. The appropriate tool for making that comparison is the Akaike Information Criteria (AIC). We compared a model of the functional form shown in equation (1) to one in which the rate decreases exponentially,

$$\frac{d[Mo_{36}]}{dt} = a * \exp(-b(t-c)). \qquad (2)$$

The first model, which fit the first derivative of the sigmoid curve shown in equation (1), had an AIC of -220.51. Meanwhile the second model, which fit an exponential decay to the rate, shown in equation (2), had an AIC of -206.58. We interpret the significantly lower AIC value associated with the first derivative of the sigmoidal curve as evidence that the data is more appropriately explained using the model in which the rate follows the functional form of equation (1), lending credence to our assumption that the underlying dynamics are governed by an autocatalytic process.

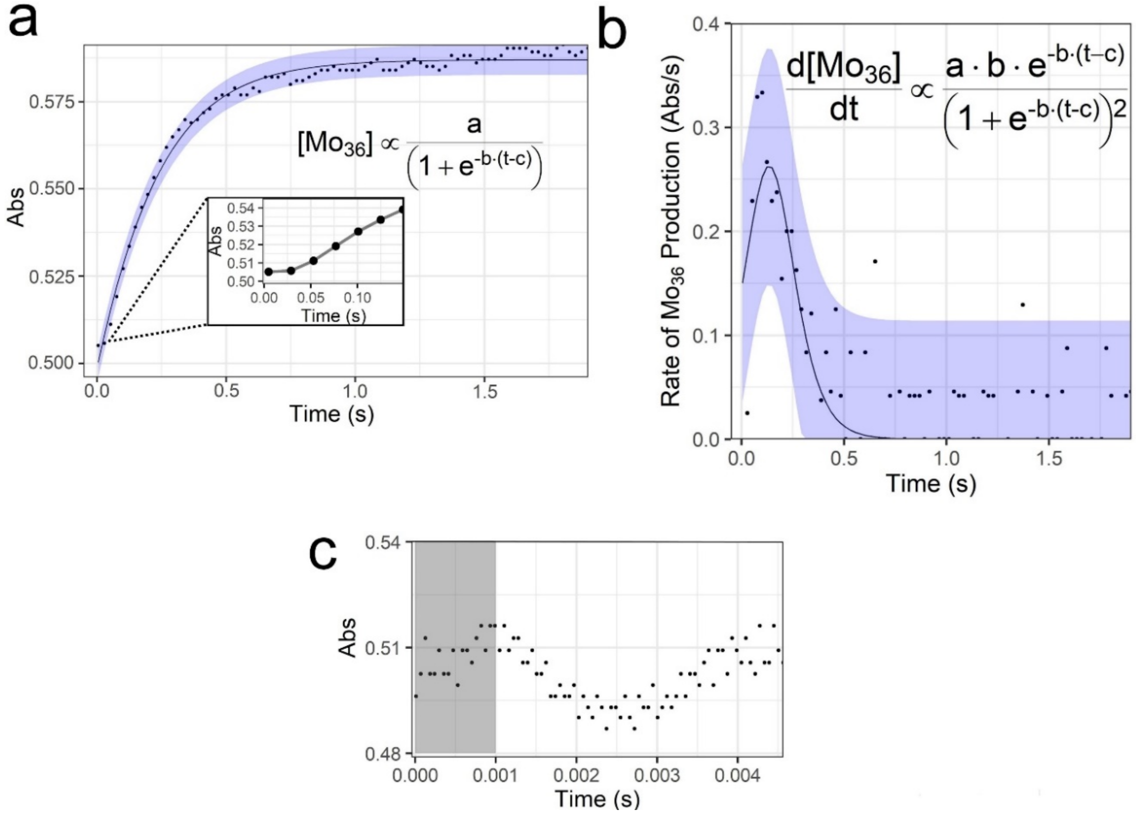

Figure S3. Formation and reaction rate of {Mo_{36}} formation. a. Concentration *vs.* time profile of {Mo_{36}} (in H_2O at 24.3 °C), initial concentrations [Mo] = 0.25 M, [H^+] = 0.047 M. The circles are experimental data points, while the line corresponds to a sigmoidal fit with the uncertainty of the fit shown in the blue region. **b.** Using the time series concentration profile we calculate the rate formation of {Mo_{36}} as a function of time using a finite difference method applied directly to the data. The first derivative of the sigmoidal fit from the concentration time series is shown with the uncertainty as well.

c. Representation of the high-resolution data collection of the same reaction mixture using 5 ms scan time highlighting the incubation period during the first stage of the reaction. The greyed-out area corresponds to the dead-time of the instrument which is ignored.

3-2: Inhibition of molecular recognition in the formation process of {Mo_{36}} species and identification of the key species.

After this first indication of underlying autocatalytic process, we carried out a series of control experiments which would allow us to comment further on the nature of the reaction networks that exist in the chemical system. Specifically, we conducted a series of experiments aiming at identifying the key species involved in the cycle and inhibiting the interaction/recognition process of the fundamental molecular synthons which take place during the autocatalytic and subsequent self-assembly process in different ways. One obvious set of parameters that affect the process is the concentration of protons and reducing environment which are directly related to the viability of the virtual building block library which is formed at the early stages of the reaction. The Supplementary Figure 4 shows that when the [H^+] > 10^{-4} M prevents the formation of the necessary building blocks and further molecular recognition from taking place. In a similar manner, if we generated a reducing environment beyond a specific range (reduction of Mo centres > 25-30 %) the molecular recognition and consequently the formation of {Mo_{36}} is dramatically diminished (Supplementary Figure 5).

In another series of control experiments, we retained the exact experimental conditions where we observe the optimum autocatalytic effect on the formation of the {Mo_{36}} cluster and aimed to inhibit the molecular recognition between constituents of the building block library. In order to achieve that we utilised molecular species able to form multiple H-bonds which could act as inhibitors, such as oxalic and trimesic acid. In this case we observed a considerable decrease of the concentration of {Mo_{36}} formed in solution as a function of the number of the available functional groups of the organic ligand able to form H-bond, see Supplementary Figure 6.

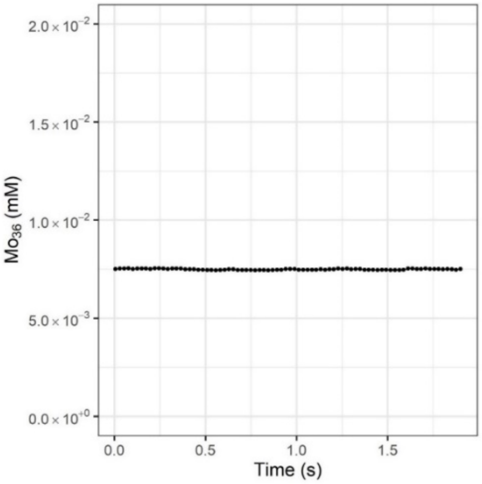

Figure S4. pH effect in the synthesis of {Mo_{36}} wheel. Inhibition of the {Mo_{36}} formation due to the disruption of the molecular interaction/recognition process. Concentration *vs.* time profile of {Mo_{36}} (in H_2O at 24.3 °C), initial concentrations [Mo] = 0.25 M, pH > 4.5. The circles represent experimental data points. The increased [H^+] concentration of the reaction mixture prohibits the initial formation of the necessary building blocks for the formation of the autocatalyst.

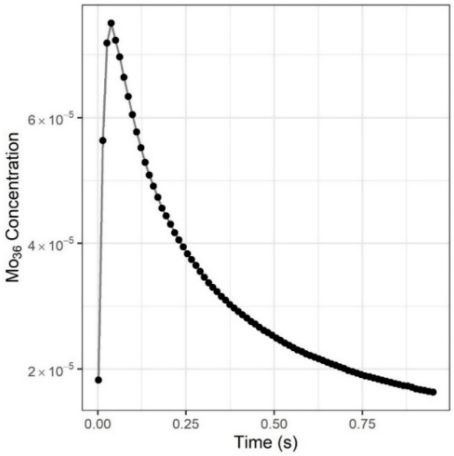

Figure S5. Effect of reducing environment in the synthesis of {Mo_{36}}. Concentration (M) *vs.* time (s) profile of {Mo_{36}} (in H_2O at 24.3 °C), initial concentrations [Mo] = 0.075 M, [H^+] = 0.1 M, [e^-] = 0.023 M. The circles are experimental data points. The over-reduction (>64 % based on Mo) of the solution rapidly decomposes the autocatalyst and consequently prevents any further recognition.

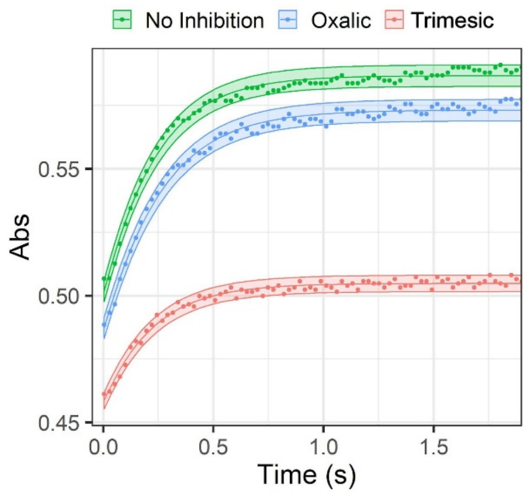

Figure S6. Inhibition of molecular recognition. Absorption *vs.* time profile of {Mo_{36}} (in H_2O at 24.3 °C), initial concentrations [Mo] = 0.25 M, [H^+] = 0.047 M, [Tri] = 0.18 M. The plot represents experimental data points while the line corresponds to the applied fitting with the uncertainty of the fit shown in the coloured regions. The molecular recognition is inhibited as a function of the number of the available H-bond sites provided by the inhibitor.

3-3: Rate increase and elimination of induction period upon seeding of the reaction with {Mo_{36}}.

Since we observed the exponential growth of the {Mo_{36}} species in solution and interception of the molecular recognition processes by disrupting the network of hydrogen bonds between the building blocks, we investigated the effect on the kinetics of the reaction by "seeding" the solution with pre-formed autocatalyst. Specifically, we demonstrated that seeding the reaction mixture with {Mo_{36}} eliminates the incubation period required to produce {Mo_{36}} and increases the initial growth rate during the exponential growth phase. The Supplementary Figure 7 represents these changes that take place during the formation of the {Mo_{36}} cluster. The additional of pre-formed {Mo_{36}} cluster eliminates the induction period and increase the initial rate of the reaction which is a further indication of the existence of an embedded autocatalytic set.

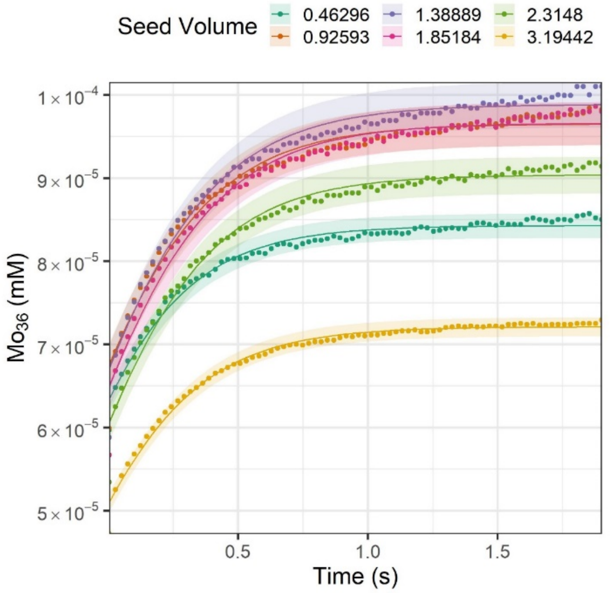

Figure S7. Effect of "seeding" using preformed {Mo_{36}}. Concentration *vs.* time profile of {Mo_{36}} (in H_2O at 24.3 °C), initial concentrations [Mo] = 0.25 M, [H^+] = 0.047 M. The points represent the concentration profile vs time of the same reaction mixture seeded with preformed {Mo_{36}} 0.0016 M.

3-4: Autocatalyst saturation

Based on our observations from the previous sections we prepared and recorded an array of different "seed" concentrations. More specifically, we injected specific amounts of preformed {Mo_{36}} cluster in the reaction mixture. Here we demonstrate that the initial rate of production increases as the seed concentration increases up to a saturation point, after which it is kinetically inhibited by the reactant concentrations.

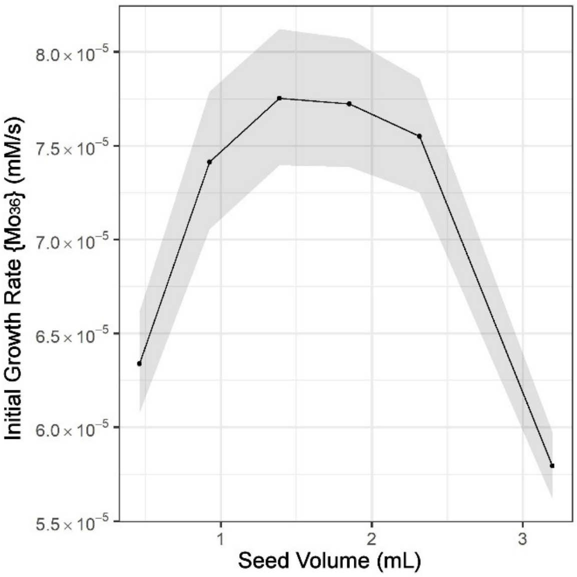

Figure S8. Kinetic saturation of {Mo_{36}} autocatalyst. The plot represents the initial formation rate of {Mo_{36}} *vs*. mL of preformed {Mo_{36}} (0.0016 M) injected in the reaction mixture (in H_2O at 24.3 °C); initial concentrations [Mo] = 0.25 M, [H^+] = 0.047 M. The experimental data points represent the initial rate of the system while the line corresponds to the applied fitting with the uncertainty of the fit shown in the grey region. An increase is observed at the beginning before reaching a short plateau (saturation) followed by decrease of the initial rates upon addition of larger amounts.

3-5 Effect of {Mo_{36}} on the {Mo_{154}} assembly

The autocatalysis and replication which takes place during the first cycle has a profound effect on the formation of the family of Mo-blues (MB) wheels via a cross-catalytic process, see Supplementary Figure 9. The effect of the embedded autocatalytic process in the minimal autocatalytic set is reflected upon the amount of the Molybdenum blue wheel species, {$Mo_{(154-x)}$} (where x = 0 – 12), formed in solution at the specific time scale as well as the observed formation rates of the reaction. More specifically, we observe an obvious acceleration of the {$Mo_{(154-x)}$} wheel formation in the presence of small amounts of the template {Mo_{36}} and even more in the case where the template was used as a starting material, see Figure S21.

Additionally, the system has been investigated further in relation to other the parameters that affect the formation of the MB wheels. The family of {$Mo_{(154-x)}$} wheels can be formed within the pH range of 1.3 to 5.0. Even though the structural motif remains the same the nuclearity decreases as a function of increased pH value due to elimination of {Mo_2} units from the wheel's rim. Supplementary Figure

11 shows the pH range where we have optimum synthetic conditions for the template $\{Mo_{36}\}$ cluster, we observe faster evolution of the band in the visible region (740 nm) which corresponds to the formation of the MB wheels.

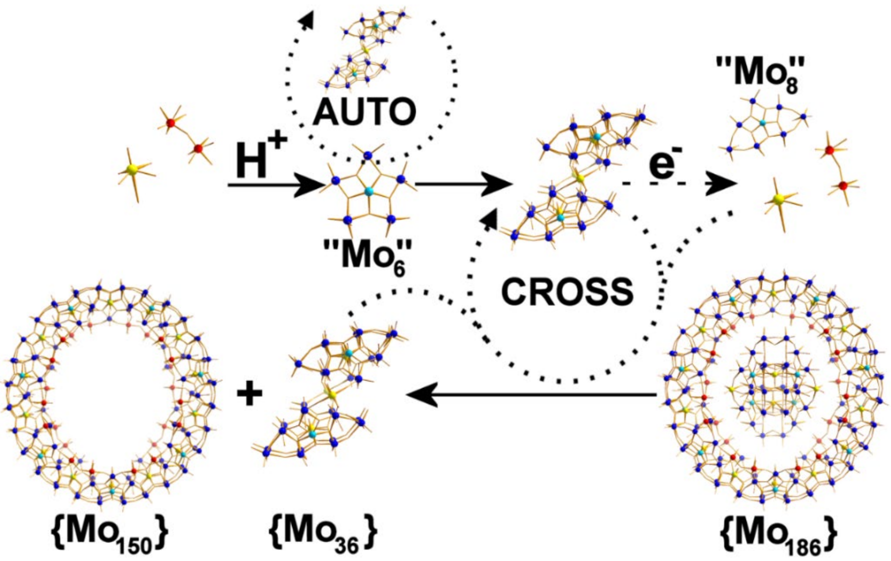

Figure S9. Autocatalytic and cross-catalytic cycles in the synthesis of $\{Mo_{154-x}\}$ wheels. Representation of the minimal autocatalytic set. The ball-and-stick representation highlights the initial constituents of the building block library which consists of $\{Mo_8\}$ as well as $\{Mo_2\}$ and $\{Mo_1\}$ groups. Initially, the $\{Mo_{36}\}$ cluster is formed by 4 x $\{Mo_8\}$ and 2 $\{Mo_1\}$ units. The formation of the "pentagonal" building blocks ("Mo_6") involving a 7-coordinate molybdenum centre (cyan) are crucial for the further assembly of the characterised species. Finally, the $\{Mo_{36}\}$ cluster templates the formation of the family of molybdenum blue wheels. The $\{Mo_8\}$ fragments are bridged together by 12 $\{MoO_2(H_2O)\}^{2+} \equiv \{Mo_2\}$ groups. $\{Mo_1\}$ = yellow; $\{Mo_2\}$ = red; $\{Mo_8\}$ = blue.

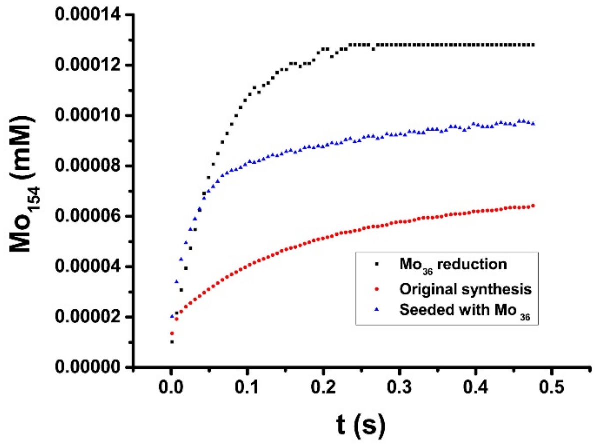

Figure S10. {Mo_{36}} effect in the synthesis of {Mo_{154-x}} wheel. Representation of the absorption band at 740 nm as a function of the time for the formation of {Mo_{154-x}} wheels at pH = 1.7 (in H_2O at 24.3 °C) during the original synthesis (red line), seeded reaction mixture with preformed {Mo_{36}} (2.6 x 10^{-3} M) cluster (blue line) and reduction of preformed {Mo_{36}} (2.6 x 10^{-3} M) cluster, (black line). Initial concentrations of the solutions used were [Mo] = 0.16 M, [H^+] = 0.33 M. The amount of $Na_2S_2O_4$ (reducing agent) used reduced approx.. 20% of molybdenum centres. The curves are experimental data points. In the case of the reduction of preformed {Mo_{36}} (black line), saturation of the detector observed after 0.25 seconds.

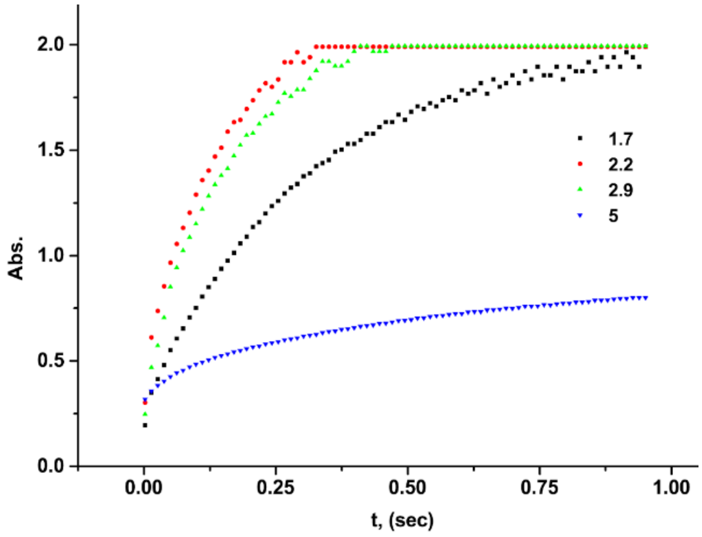

Figure S11. pH effect in the synthesis of $\{Mo_{154-x}\}$ wheel. Representation of the absorption band at 740 nm as a function of the time for the formation of $\{Mo_{154-x}\}$ wheels at different pH values (in H_2O at 24.3 °C); initial concentrations of stock solutions [Mo] = 0.16 M, $[H^+]$ = 1 M (diluted accordingly), $[e^-]$ = 0.025 M. The curves are experimental data points representing the concentration of the $\{Mo_{154}\}$ generated produced over time. At pH values of 2.2 and 2.9 saturation of the detector observed after 0.25 seconds.

In a similar fashion, the system has been investigated also in relation to the reducing environment which also affect the formation of the MB wheels. Figure S12 shows that the optimum percent of reduced molybdenum centres which induces the formation of the $\{Mo_8\}$ secondary building blocks, lies between the values ~10-20 % as we observe faster evolution of the band in the visible region (740 nm) which corresponds to the formation of the MB wheels. Any value below 9% of reduction does not provide enough concentration of necessary building blocks which results in slow formation of the wheels. On the other hand, over reduction of the system (>40 %) leads to the destruction of the $\{Mo_2\}$ and $\{Mo_1\}$ which are necessary components for the assembly of the nanosised wheels.

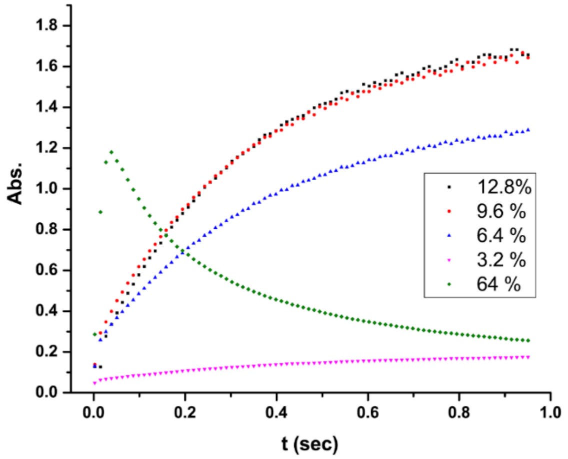

Figure S12. Effect of reducing environment in the synthesis of $\{Mo_{154-x}\}$ wheel. Representation of the absorption band at 740 nm as a function of the time for the formation of $\{Mo_{154-x}\}$ wheels at different reducing environments (in H_2O at 24.3 °C); initial concentrations of stock solutions [Mo] = 0.16 M, $[H^+]$ = 0.5 M, $[e^-]$ = 0.04 M (diluted accordingly). The curves are experimental data points. Excessive reduction of the Mo content (64 %) induces the fast formation of the necessary building blocks at the very early stages of the reaction (<0.1 sec) but rapidly decompose to over-reduced molybdenum oxide solids which prevents the formation of the $\{Mo_{154-x}\}$ family.

4: Synthesis of $\{PMo_{12}\}\subset\{Mo_{124}Ce_4\}$ wheel demonstration of $\{Mo_{12}\}$ Autocatalysis

Using the same techniques and approaches that we demonstrated for $\{Mo_{36}\}$, we also provide evidence that $\{Mo_{12}\}$ it autocatalytic. The timescales associated with exponential growth of $\{Mo_{12}\}$ is much shorter than that of $\{Mo_{36}\}$, meaning that it is harder to resolve and identify the phenomenon using the experimental platform. Nonetheless we are able to observe similar behaviour.

4-1: Synthesis and effect of $\{Mo_{12}\}$ cluster on the assembly of $\{PMo_{12}\}\subset\{Mo_{124}Ce_4\}$ wheel

The new Keggin templated wheel, $(C_6H_{14}N_2O_4S_2)_4K[H_{16}Mo^{VI}_{100}Mo^{V}_{24}Ce_4O_{376}(H_2O)_{56}(PMo^{VI}_{10}Mo^{V}_2O_{40})(C_6H_{12}N_2O_4S_2)_4]\cdot200H_2O$, was prepared as follows:

A solution of $CeCl_3.6H_2O$ (6.0 g, 16.4 mmol) in H_2O (300 mL) was quickly added under stirring to an aqueous solution of K_2MoO_4 (5.9 g, 24.8 mmol) in H_2O (300 mL). The yellow precipitate $Ce_2O_3\cdot7MoO_3\cdot6H_2O$ was collected by filtration after 30 min, washed with ice-cold H_2O, and dried at 120 °C for 5 h (yield: 7.2 g; IR (KBr; 1700-500 cm^{-1}): 1625 (m), 1384 (m), 937 (m), 859 (s), 761 (s), 699 cm^{-1}(m).

To an aqueous solution made of H_2O (50 mL) and 1M $HClO_4$ (4.5 mL) of $Ce_2O_3{\cdot}7MoO_3{\cdot}6H_2O$ (0.2 g), an aqueous solution (0.4 mL) of $N_2H_4{\bullet}2HCl$ (10 g/L), cysteine (14.5 mg, 0.12 mmol) and $H_3PMo_{12}O_{40}{\cdot}xH_2O$ (20 mg) were added. The solution was heated with medium stirring in a 50-mL Erlenmeyer flask (wide-necked; covered with a watch glass) at 90° C for 5 h. The resulting deep-blue solution was then cooled to room temperature and kept in the flask for 12 h during which period of time a small amount of dark blue precipitate was formed and removed by filtration. The filtrate was kept in an open 50-mL Erlenmeyer flask for 2 weeks. The deep-blue plate-like crystals were collected by filtration, washed with ice-cold H_2O, and dried under inert atmosphere over $CaCl_2$, yield: 0.032g (15.2 % based on Mo). Characteristic IR bands (KBr; 1800-500 cm^{-1}): 1725 (w; *n* (C=O)), 1608 (s, $n_{asym}(CO2^{-})$ + d(H2O)), 1491 (m, d(NH_3^+)), 1407 (m), 1342 (m), 1251 (w), 1127 (w), 1055 (m), 964 (m; *n* (Mo=O)), 789 (s), 633 (s), 555 (s) cm^{-1}. Elemental analysis, *calc.*: Ce 2.08, Mo 48.52, K 0.15, P 0.12, S 1.91, C 2.14, N 0.83 H 2.37 %; Found Ce 2.44, Mo 48.10, P 0.11, K 0.18, S 1.91, C 2.03, N 0.83 H 1.71 %.

Single-crystal X-ray structural analysis reveals that **1** crystalises in the space group C 2/m and features a nanoring $\{Mo_{124}Ce_4\}$, composed of 12 $\{Mo_8\}$ units, 8 $\{Mo_2\}$ units, 12 $\{Mo_1\}$ units, 4 $\{Ce(H_2O)_5\}$ units and 4 Cysteine, with a $\{PMo_{12}\}$ Keggin cluster trapped in the centre (Supplementary Figure 13). The four Ce^{3+} ions are distributed symmetrically on the two ends of both the upper and lower rims of $\{Mo_{124}Ce_4\}$, making the whole wheel exhibit a configuration with C_2 symmetry. Therefore, the wheel displays a relatively symmetric structure with an oval shaped opening with outer and inner ring diameter of about 29 and 19 Å, respectively. Additionally, there are four dimerised cysteine molecules which exhibit S-S bonds and each one of them is bridging two $\{Mo_2\}$ units located on the upper and lower rims via the two antipodal carboxylate groups.

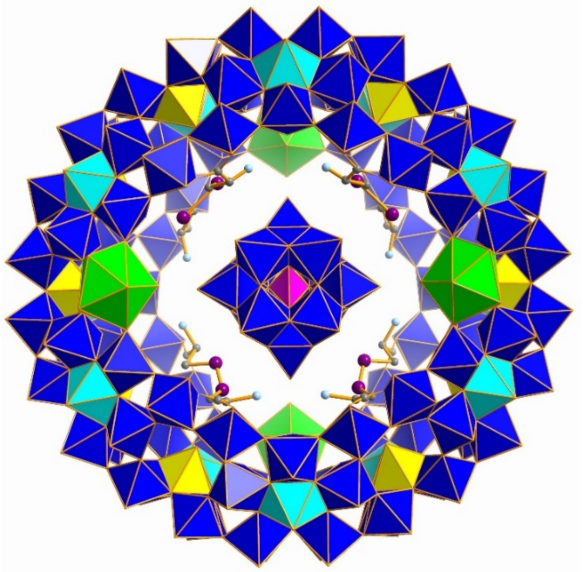

Figure S13. View of the molecular structure of 1. {Mo_1}, yellow polyhedron; {Mo_8}, blue polyhedron with central pentagonal units in cyan polyhedron; Ce, green polyhedron; O, red; C, grey; S, violet; P, pink; N, light blue. The cysteine molecules are presented in ball and stick model.

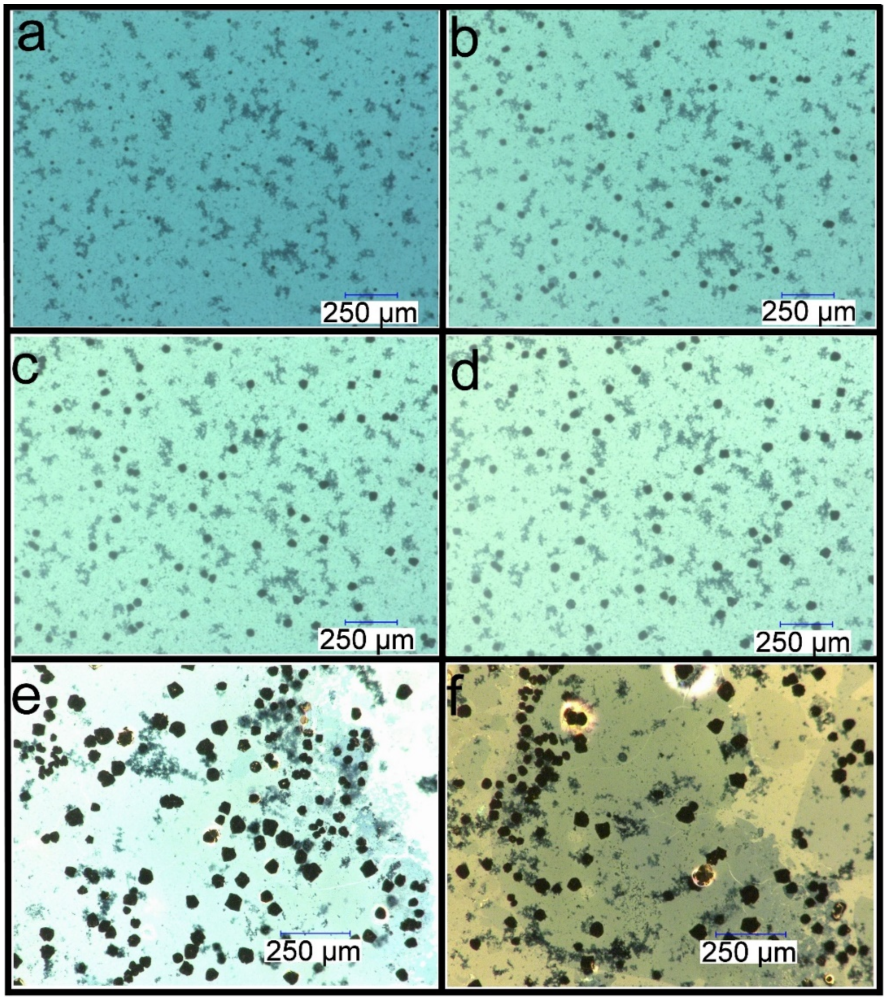

Figure S14. Crystallization of $\{PMo_{12}\}\subset\{Mo_{124}Ce_4\}$. The following scaled down synthetic procedure was used for the recording purposes of the crystallisation; $7MoO_3.Ce_2O_3$ (200 mg) was dispersed in 8 mL H_2O, then 1.8 mL 1M HClO4, 0.6 mL 0.5 M cysteine and $H_3PMo_{12}O_{40}$ (20 mg) were added. The mixture was heated at 90 °C for 1-2 h and filtered while hot. The hot filtrate was placed immediately in petri dishes (t=0). Observation of the $\{PMo_{12}\}\subset\{Mo_{124}Ce_4\}$ crystallization at: a. 20 min; b. 40 min; c. 60 min; d. 90 min; e. 120 min and f. 150 min.

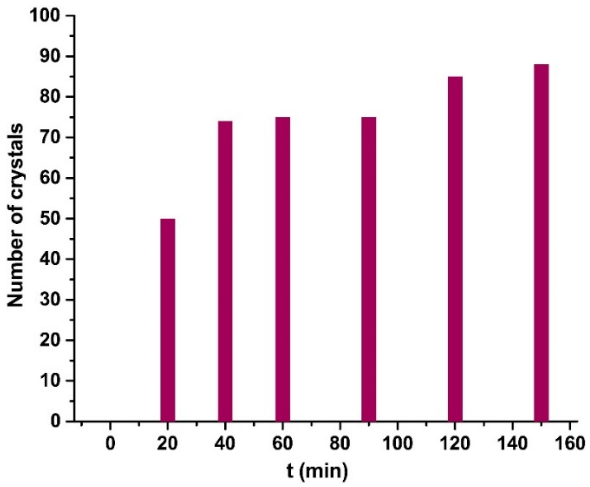

Figure S15. Number of $\{PMo_{12}\}\subset\{Mo_{124}Ce_4\}$ crystals. Approximate number of $\{PMo_{12}\}\subset\{Mo_{124}Ce_4\}$ crystals observed at: a. 20 min; b. 40 min; c. 60 min; d. 90 min; e. 120 min and f. 150 min.

The autocatalytic phenomenon which takes place during the first stages of the assembly process has a similar effect on the formation of the $\{PMo_{12}\}@\{Mo_{124}Ce_4\}$ via a cross-catalytic process, see Figure S16. The effect of the embedded autocatalytic process in the minimal autocatalytic set is reflected upon the amount of the Molybdenum blue wheel shaped species, $\{Mo_{124}Ce_4\}$, formed in solution at the specific time scale. Also in this case, we observed an obvious acceleration of the $\{Mo_{124}Ce_4\}$ wheel formation in the presence of small amounts of the template $\{PMo_{12}\}$ and even more in the case where the template was used as a starting material, see Figure S19.

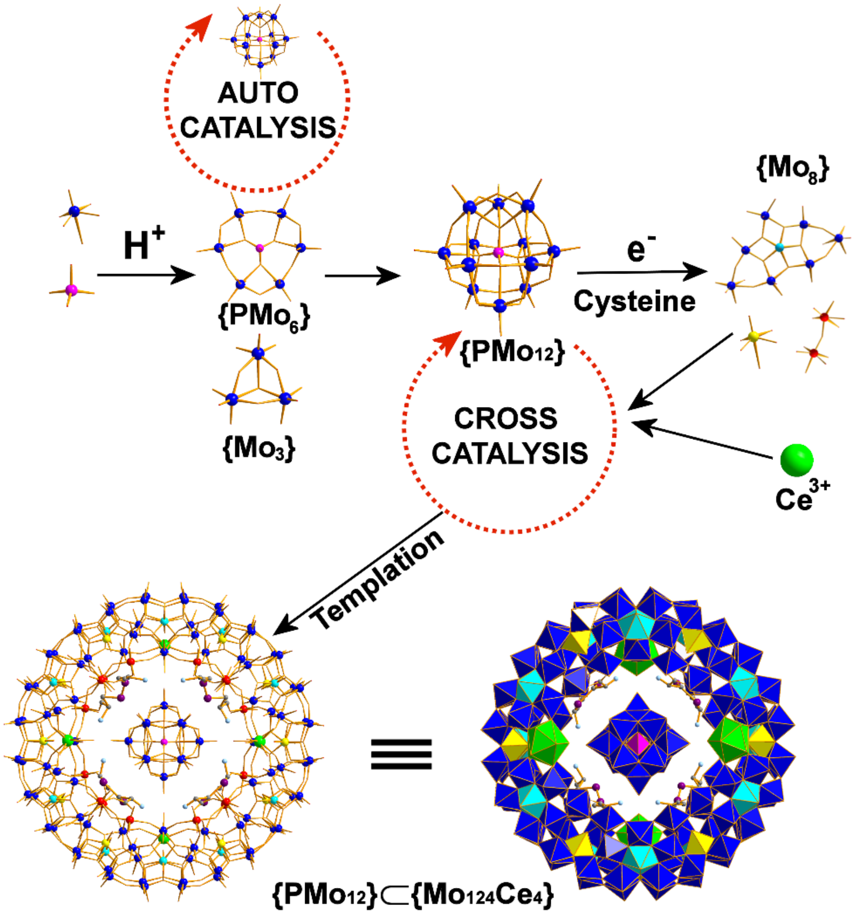

Figure S16. Autocatalytic and cross-catalytic cycles in the synthesis of {Mo_{124}} Wheel. Representation of the minimal autocatalytic set which induces the formation of the Keggin templated {Mo_{124}} Wheel.

4-2: Sigmoidal traits of product/time curve for {PMo_{12}}

In a similar fashion, the experimental data were collected and processed using the ProData software supplied by Applied Photophysics. The sample containers were loaded with freshly prepared solutions of $Na_2MoO_4{\cdot}2H_2O$, (0.7 M) and HCl (0.06 M) and were mixed in equal volumes and the absorbance corresponding to the formation the {Mo_{12}} cluster (440 nm) was monitored as a function of the time. The scan rate was set to t=1 sec.

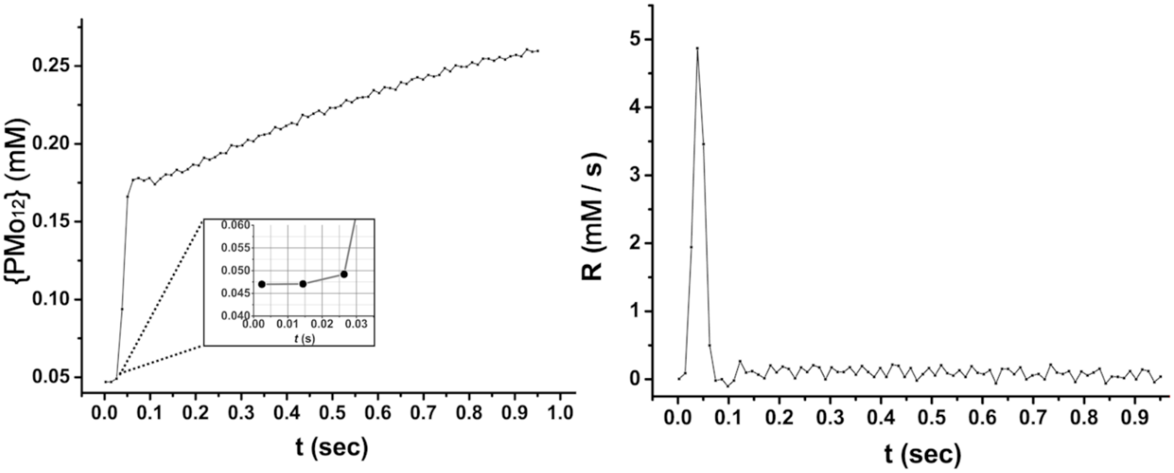

Figure S17. Formation and reaction rate of $\{PMo_{12}\}$ formation. Absorption *vs.* time profile of $\{PMo_{12}\}$ (in H_2O at 24.3 °C), initial concentrations [Mo] = 0.7 M, $[H^+]$ = 0.06 M. The squares are experimental data points. The inset highlights the lag time observed at the early stages of the reaction. The curve represents experimental data at 440 nm. Unfortunately due to the time resolution we are not able to generate robust fits to this data. Using the time series concentration profile we calculate the rate of rate of formation of $\{PMo_{12}\}$ as a function of time by using a finite difference method applied directly to the data.

4-3: Template / recognition effect in the formation of $\{Mo_{12}\}$

The previously reported procedure for the synthesis of $\{PMo_{12}\}$ species ($Na_2HPMo_{12}O_{40}$), involves the addition of the template (H_3PO_4) in a molybdate aqueous solution while the pH value is adjusted by the addition of a secondary source of protons (in this case $HClO_4$). In this case we tested the hypothesis of an underlying template / recognition effect in this system by using a template of tetrahedral geometry which carries different charge. The control experiments involved the use either of $HClO_4$ or H_2SO_4 in the absence of PO_4^{3-} anions. As shown in the the Supplementary Figure 14, only in the case where PO_4^{3-} anions are present in the reaction mixture we were able to observe a very fast growth of the Keggin species in solution in such a short period of time.

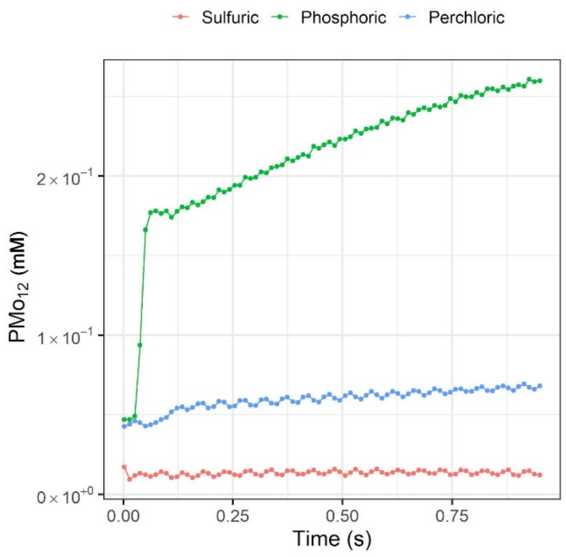

Figure S18. Template and recognition effect in the synthesis of {PMo_{12}}. Concentration *vs.* time profile of {PMo_{12}} (in H_2O at 24.3 °C), initial concentrations [Mo] = 0.7 M, [H^+] = 0.06 M. (a) Reaction of Mo in the presence of H_3PO_4 leading to the formation of {PMo_{12}} Keggin; (b) Control experiment using the same concentration of $HClO_4$ and (c) H_2SO_4 respectively. The curve represents experimental data at 440 nm.

4-4: Rate increase and elimination of induction period upon seeding of the reaction with {Mo_{12}}.

Based on the observation of exponential growth of the {Mo_{12}} species in a comparable manner to the {Mo_{36}} template (see section SI-3-3), we carried out a series of experiments involving "seeding" of the reaction mixture with preformed {Mo_{12}} cluster. The addition of preformed {Mo_{12}} species caused the gradual elimination of the induction period (Figure S15) observed initially and increases the initial growth rate during the exponential growth phase. The Figures S15 and S16 show these changes that take place during the formation of the {Mo_{12}} cluster.

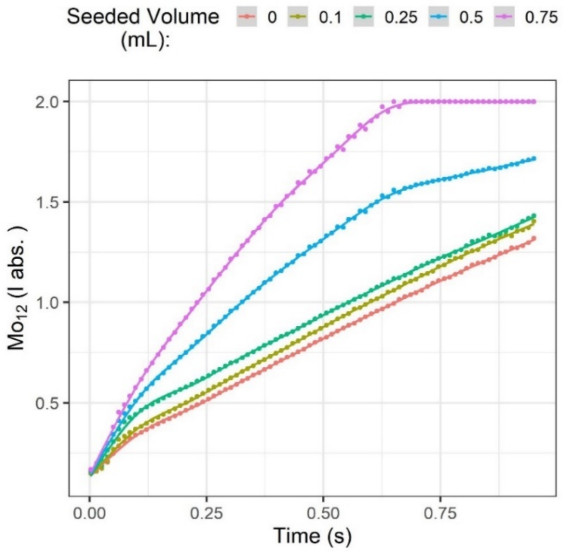

Figure S19. Effect of “seeding” with preformed {PMo_{12}}. Absorption *vs.* time profile of {Mo_{12}} (in H_2O at 24.3 °C), initial concentrations [Mo] = 5.6 x 10^{-4} M. The curves are experimental data points under different conditions (Black, original synthesis); Coloured lines, Seeded reaction mixture with 0.1 – 2 mL of preformed {Mo_{12}} (0.01 M) autocatalyst. Observation of the gradual elimination of the induction period.

4-5: Kinetic saturation of {Mo_{12}} synthesis

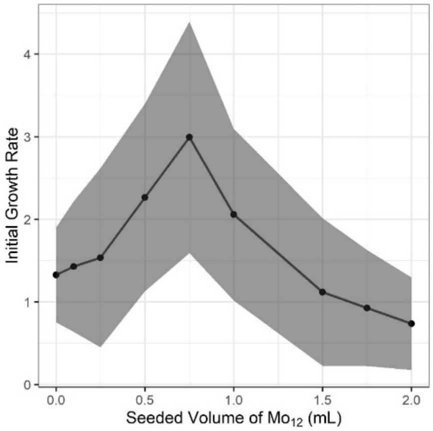

Figure S20. Kinetic saturation of {PMo_{12}} autocatalyst. Effect of the seeding experiment on the initial rates of the reaction as a function of the [PMo_{12}] injected in the reaction mixture (in H_2O at 24.3 °C). A small increase is observed at the beginning before reaching a maximum (saturation) followed by an abrupt drop of the initial rate.

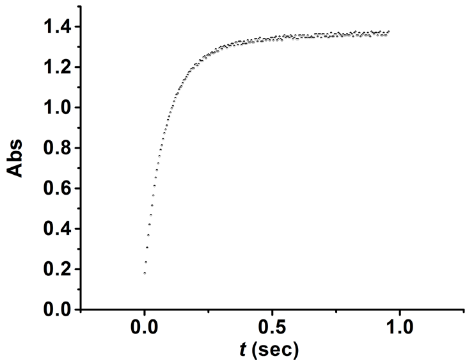

Figure S21. Reduction of {PMo_{12}} reaction mixture. Concentration *vs.* time profile of the {Mo_{154-x}} formation (in H_2O at 24.3 °C), initial concentrations [Mo] = 0.7 M, [H^+] = 0.047 M, [e^-] = 0.013 M. The curve represents experimental data at 740 nm.

5: Isotopic effects

To verify the importance of the development of a network of hydrogen bonds between the species that act autocatalytically and the primary building blocks we monitored the formation of the {Mo_{36}}, {Mo_{12}} and {Mo_{154}} wheel in deuterated solvent. The importance of the hydrogen bond formation and molecular recognition has already been shown with the inhibition experiments discussed above; additionally, we observed the isotopic effect on the formation process of the autocatalytic species and the effect it had on the reaction rates. As it was expected the gradual increase of the deuterium content is directly associated with the slower rate of the reaction under investigation.

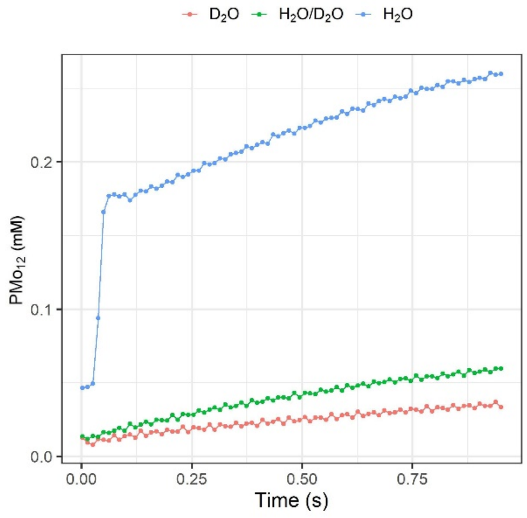

Figure S22. Isotopic effect in the synthesis of {PMo_{12}}. Absorption *vs.* time profile of the Keggin formation (in H_2O, (cyan); D_2O, (orange) and D_2O:H_2O, (green) at 24.3 °C), initial concentrations [Mo] = 0.7 M, [H^+] = 0.047 M. The curve represents experimental data at 490 nm.

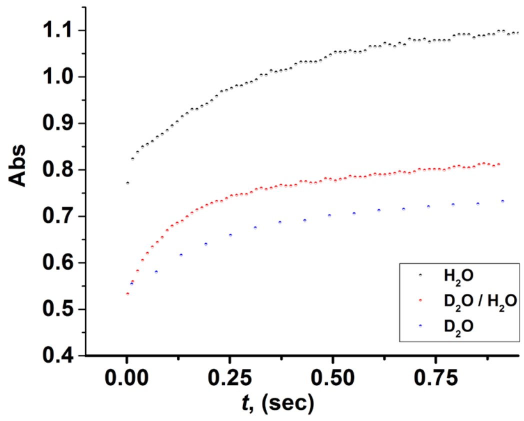

Figure S23. Isotopic effect in the synthesis of {Mo_{36}}. Absorption *vs.* time profile of the Mo_{36} formation (in H_2O, (black); D_2O, (blue) and D_2O:H_2O, (red) at 24.3 °C), initial concentrations [Mo] = 0.7 M, [H^+] = 0.047 M. The curve represents experimental data at 390 nm.

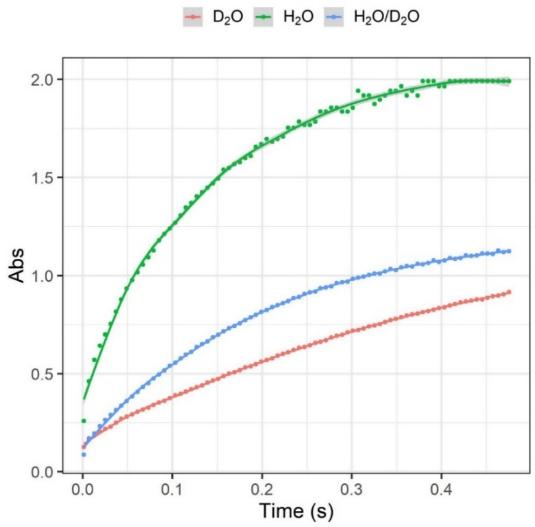

Figure S24. Isotopic effect in the synthesis of $\{Mo_{154}\}$. Absorption *vs.* time profile of the Mo_{154} wheel formation (in H_2O, (green); H_2O / D_2O, (blue) and D_2O, (red) at 24.3 °C), initial concentrations [Mo] = 0.7 M, $[H^+]$ = 0.047 M, $[e^-]$ = 0.047 M. The curve represents experimental data at 740 nm.

SI-6: Stochastic Kinetic Model

The simulations were developed using a implementation of the Gillespie algorithm written in Julia (v1.0 later) with the packages, dataframes, CSV, combinatorics, JSON, and Random and R (V3.5 or later for analysis) with the packages ggplot2, dplyr and tidyr. The analysis was done using R via a Jupyter notebook and the full code and data for the simulations are available.

The formation of Mo nano-structures can be simulated using a kinetic Monte Carlo approach, however to the best of our knowledge such a model has never been developed or implemented. A schematic representation of this algorithm is shown in Figure S25.

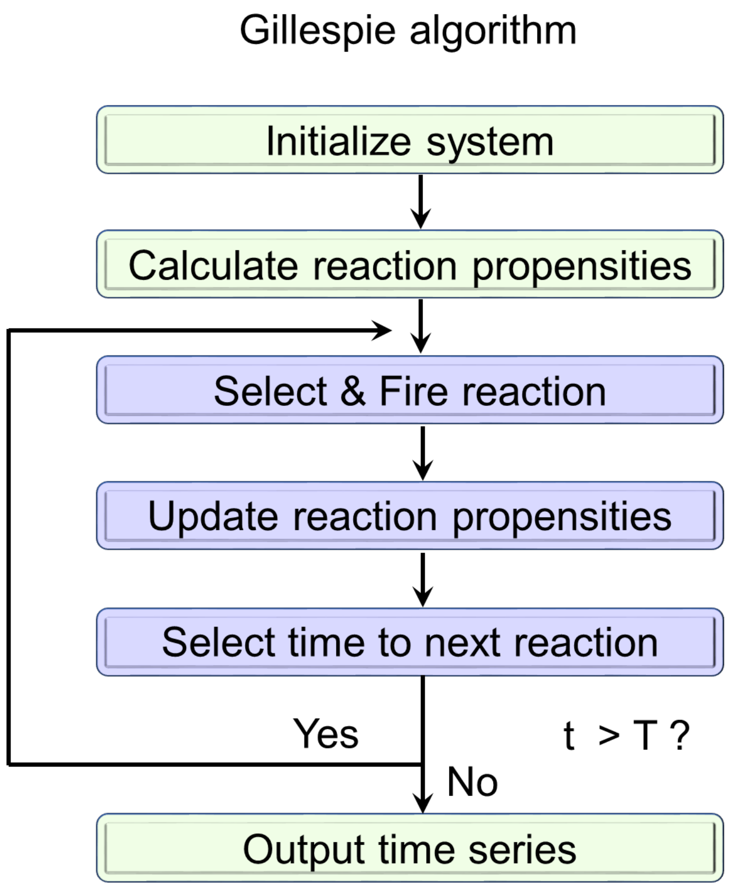

Figure S25. Gillespie Algorithm.

This technique represents all reactions as either uni-molecular (in the case of degradation, A → B+ C) or bimolecular (in the case of synthesis, A +B → C). Here we develop this model explicitly.

6-1: Model description

In this model structures and intermediates form as the products of bimolecular reactions between building blocks and other intermediates. We assume that all molecules (besides $\{Mo_1\}$) can degrade into component parts. We initialized the system with 10^6 Mo_1 molecules. Monomers can combine to form different Mo_2 species, the corner bonded dimer and the reduced edge bonded dimer. These two different dimers play different roles in the assembly dynamics, namely the corner bonded dimers can participate in future aggregation to form $\{Mo_6\}$ and other intermediates, while the edge bonded dimers cannot. The edge-bonded dimers can either degrade or bond with $\{Mo_6\}$ and other edge bonded dimers, but not with other $\{Mo_1\}$ species. The relative rate of formation of these two different dimer species is set by the parameter (D) which is the ratio between the rate constant for the formation of edge-bonded dimers to corner bonded dimers.

The following nano-structures form in the model: $\{Mo_{36}\}$, $\{Mo_{132}\}$ (Ball), $\{Mo_{154}\}$ (Wheel), as well as intermediate combinations of those structures. The effect of templating is included in this model. Templates host intermediate compounds and act to enhance the net rate of bimolecular reactions with those intermediates. Templated reactions are decomposed into three separate steps. The first is a bimolecular reaction between a reactant and the template to form complex, followed by another bimolecular reaction between the bound complex and the other reactant. The product of these two steps is a complex of the template and the reactant, which then can then under-go a unimolecular reaction to dissociate, however this complex could also undergo another bimolecular reaction to form a larger product complex before dissociating. The reaction rate constant of bimolecular reactions by a factor of k_{Mo36} when one of the reactants is bound to a template, and the unimolecular dissociation rate constant is the same as all other unimolecular reaction constants, 1.0. A schematic representation of this model is shown in figure 3 of the main text, in which black lines represent mass flow, and blue lines represent the effect of templates. To characterize the formation of giant molybdenum nano-structures, simulations were run using different sets of bimolecular rate constants. We first modeled the system by inhibiting templates ($k_{Mo36} = 0$) and varying the rate constant for all bimolecular reactions (k_f) and for three different values of the dimerization ratio (D). In supplementary figure 26 we show how the average mass of molecules and in supplementary figure 27 the number of unique species changes with these parameters.

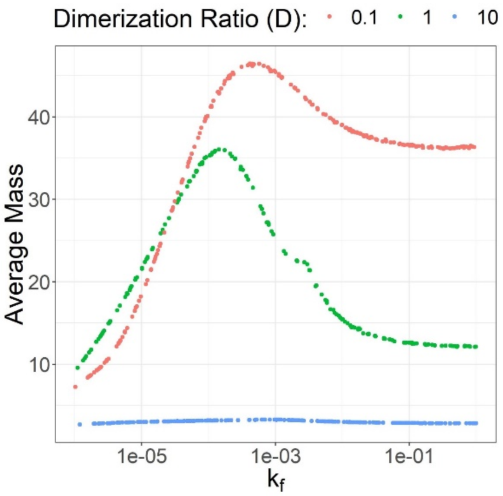

Figure S26. Average molecular mass against the forward reaction rate in the absence of templating effects. When the model does not include the effect of templates, the average molecular weight is constrained. This is a consequence of the fact that high molecular weight species do not form robustly. Each point in this figure the time averaged abundance from a single simulation and 300 simulations are shown.

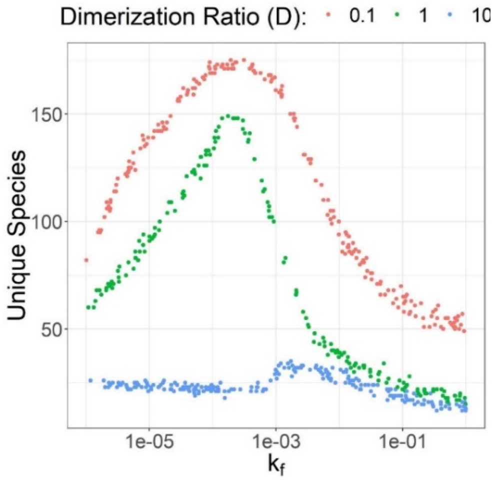

Figure S27. Steady state abundance of unique species vs the forward reaction rate. The total number of unique species is also limited by the in the absence of templates. Each point in this figure the time averaged abundance from a single simulation and 300 simulations are shown.

Under these conditions the only nano-structure which formed was {Mo_{36}}, albeit in relatively low abundance. Based on these results we next investigated the effect of templating for the formation of {Mo_6} (templated by the {Mo_{36}}) and the formation of {Mo_{154}} (also templated by {Mo_{36}}), as well as the effect of varying the dimerization ratio (D), results are shown in supplementary figure 28.

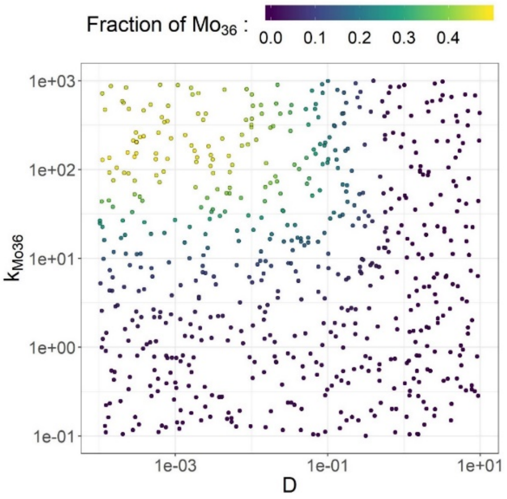

Figure S28. The steady state abundance of {Mo_{36}} is shown for various values of the dimerization ratio D, and the templating efficiency k_{Mo36}. The brighter yellow colours correspond to higher

abundance of {Mo_{36}}. Most of the parameter space does not give rise to the autocatalyst. Each point in this figure the time averaged abundance from a single simulation and 1000 simulations are shown.

We found that while including the effect of templating did result in dramatic increase in the formation of {Mo_{36}} (both in rate and in steady state abundance), it did not ensure the formation of {Mo_{154}} or {Mo_{132}}. While intermediate compounds between the Mo_{36} and the {Mo_{154}} or {Mo_{132}} formed readily, those intermediates always degraded before forming complete structures, resulting in many "frustrated attempts." This limitation cannot be easily overcome by increasing the stability of all intermediates, which only serves to preferentially increase the abundance of smaller mass intermediates, effectively trapping building blocks `down-stream.'

To overcome the frustrated formation of larger structures without *fine-tuning* the model, an additional feature was included. We assumed that the rate constant for bimolecular reactions between intermediates increased for molecules which are closer to being completed structures. This could, for example, represent the fact that a nearly complete structure serves to coordinate building blocks, pulling them into gaps in the structure. We implemented this using a linear function in the number of building blocks in the molecule with a slope of k_{nano}. Including this feature in the model results in the robust formation of larger structures over a range of parameters, without fine-tuning individual rate constants. The mass fraction of the various nano-structures is shown in supplementary figure 29, where the color corresponds to the mass fractions, the dimerization ratio is shown on the horizontal axis, and k_{nano} is shown in the vertical axis.

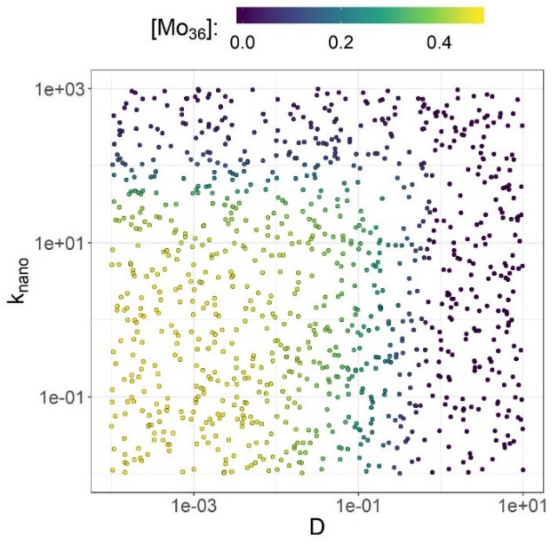

Figure S29. Mass fraction of {Mo_{36}} as a function of the dimerization rate, and the coordination term k_{nano}. Higher values of k_{nano} reduce the steady state abundance of {Mo_{36}} by shifting mass to the larger nano-structures and their intermediates. Each point in this figure the time averaged abundance from a single simulation and 2000 simulations are shown.

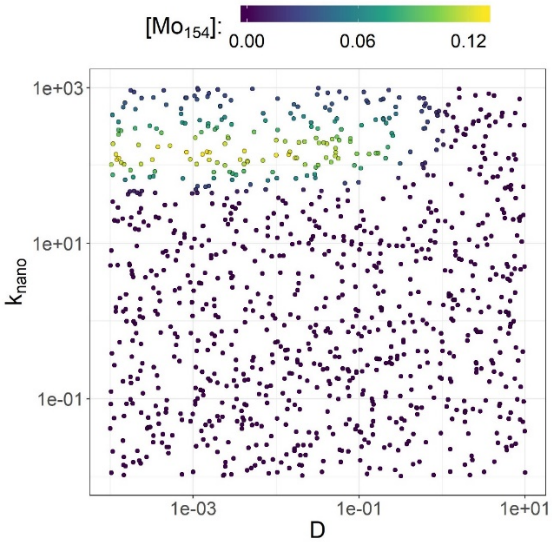

Figure S30. Mass fraction of {Mo_{154}} as a function of the dimerization rate, and the coordination term k_{nano}. Higher values of k_{nano} increase the steady state abundance of {Mo_{154}} by biasing the formation of complete nano-structures over those of incomplete ones. However, increasing the rate too much inhibits the autocatalytic function of the {Mo_{36}} by drawing away the Mo_6 building blocks, resulting in

lower abundances of {Mo_{36}} and {Mo_{154}}. Each point in this figure the time averaged abundance from a single simulation and 2000 simulations are shown.

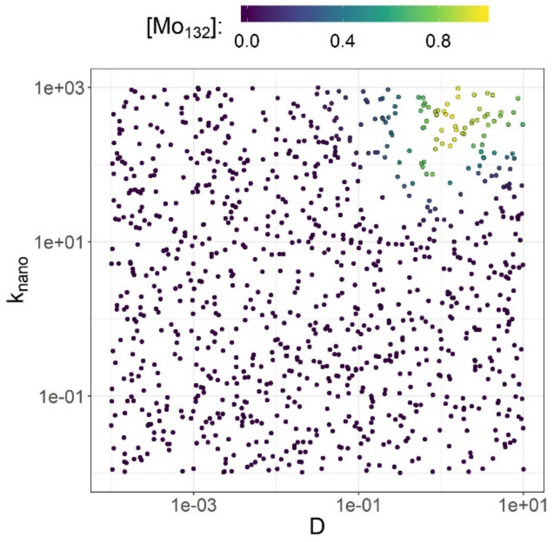

Figure S31. Mass fraction of {Mo_{132}} as a function of the dimerization rate, and the coordination term k_{nano}. Higher values of k_{nano} increase the steady state abundance of {Mo_{132}} by biasing the formation of complete nano-structures over those of incomplete ones, but the formation is limited by dimerization ratio. Each point in this figure the time averaged abundance from a single simulation and 2000 simulations are shown.

5-2: Kinetic Model Results, Predictions and comparison to experiment

By incorporating these features, the model recovers the dynamical characteristics of the experimental system. For example, the formation of the {Mo_{154}} structure and {Mo_{132}} structure is sensitive to the relative rate of dimerization, which is known to be controlled by the reduction of the solution. For more oxidised solutions the (lower relative dimerization rates) the {Mo_{154}} wheel forms in high yields until a critical point above which the wheel cannot form due to a lack of corner bonded dimers (see main text figure 3). As the rate of edge bonded dimer formation increases above this critical point, the net yield of the {Mo_{132}} in simulations rapidly increases until the formation of pentagonal units is affected at which point the yield falls quickly to zero (see main text figure 3). This phenomenon is observed by the progressive reduction of the solution in the physical experiments. Our model also recovers essential features of the formation of {Mo_{154}}, namely the autocatalytic nature of the {Mo_{36}} template. In typical simulations the abundance of Mo_{154} remains 0 for a time followed by a brief period of

exponential growth due and subsequent saturation (see Figure S32). This feature is also seen in experimental data when the solution is not seeded with {Mo_{36}}.

Our model also allowed us to explore the dynamical consequences of different assumptions surrounding the formation of molybdenum nano-structures. For example, it has been conjectured that {Mo_{36}} templates the formation {Mo_6} building blocks. Unfortunately, due to the time scales associated with the formation it is difficult to test this assumption experimentally. Using our model, we explored the consequences of including or excluding this dynamical feature. We found that Increasing the catalytic effect of the embedded {Mo_{36}} autocatalytic cycle resulted in higher steady state abundances for the {Mo_{154}} wheel, which does not form without this effect (see main text figure 3).

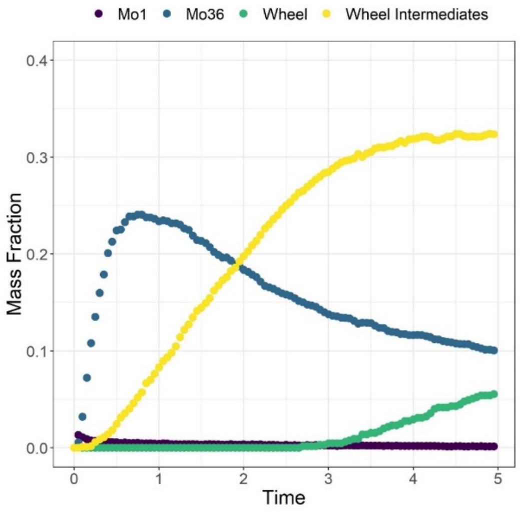

Supplementary Figure 32. Simulated time series showing the formation of {Mo_{36}} followed by the formation of the {Mo_{154}} wheel after an incubation period. Horizontal axis shows simulation time, vertical axis shows the fraction of molybdenum in structures. Blue dots correspond to {Mo_{36}}, and green dots show the {Mo_{154}} wheel, which remains zero for an extended incubation period before rising thanks to the templating effect of {Mo_{36}}. Purple dots correspond to the Mo_1 monomers which are rapidly consumed early in the simulations, while yellow dots correspond to {Mo_{154}} intermediates which included bounded {Mo_{36}} molecules as templates.

Interestingly, our model predicts that the {Mo_{154}} and {Mo_{132}} nano-structures should be able to coexist for in a very small region of the parameter space. In order to measure the coexistence, we rescaled the mass fractions of both species so that they were normalised by the highest observed value (values for both), and then defined the coexistence $\xi = \frac{2[Mo^*_{154}][Mo^*_{132}]}{[Mo^*_{154}]+[Mo^*_{132}]}$, where the concentrations of {Mo_{154}} and {Mo_{132}} have been normalised by the maximum observed value across all simulations. In Figure

S33a you can see the parameter space where the two giant nano-structures coexists, it is extremely sensitive to the dimerization ratio D, and depends on the free parameter of our model k_{nano}.

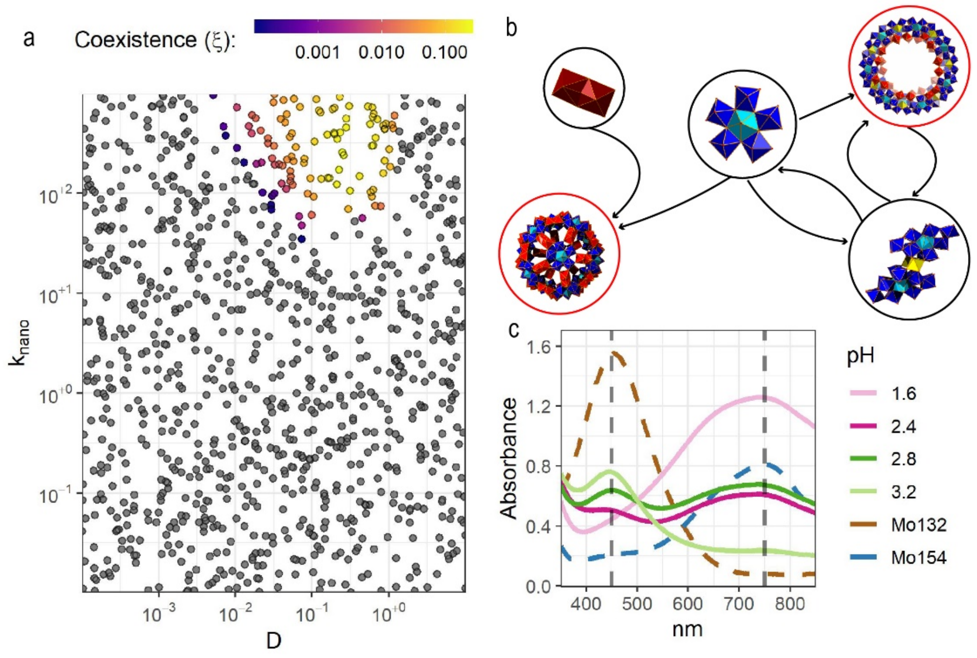

Figure S33. The model predicts that the {Mo_{154}} and {Mo_{132}} nano-structures can coexist for a narrow range of parameters. This was validated using UV-vis. The spectra presented dashed lines were obtained using crystals of preformed {Mo_{132}} Keplerate ball (brown line) and {Mo_{154}} (blue line) respectively.

We next sought to directly compare this prediction to the physical system. The gradual increase of the reduced molybdenum content up to 60% we investigated a range of pH values (1.6 – 4.2) and looked for the co-existence of the Mo_{132} and Mo_{154} nano-structures as evidenced by UV-vis absorbance. The results of those experiments are shown in Supplementary Figure 33c. At pH values of 2.4 and 2.8 we observed bands centered at 450 and 750 nm indicating the co-occurrence of the Mo_{132} and Mo_{154} nano-structures. As in the model, this coexistence is extremely sensitive to the rate of dimerization and therefore only occurs in a narrow pH range.

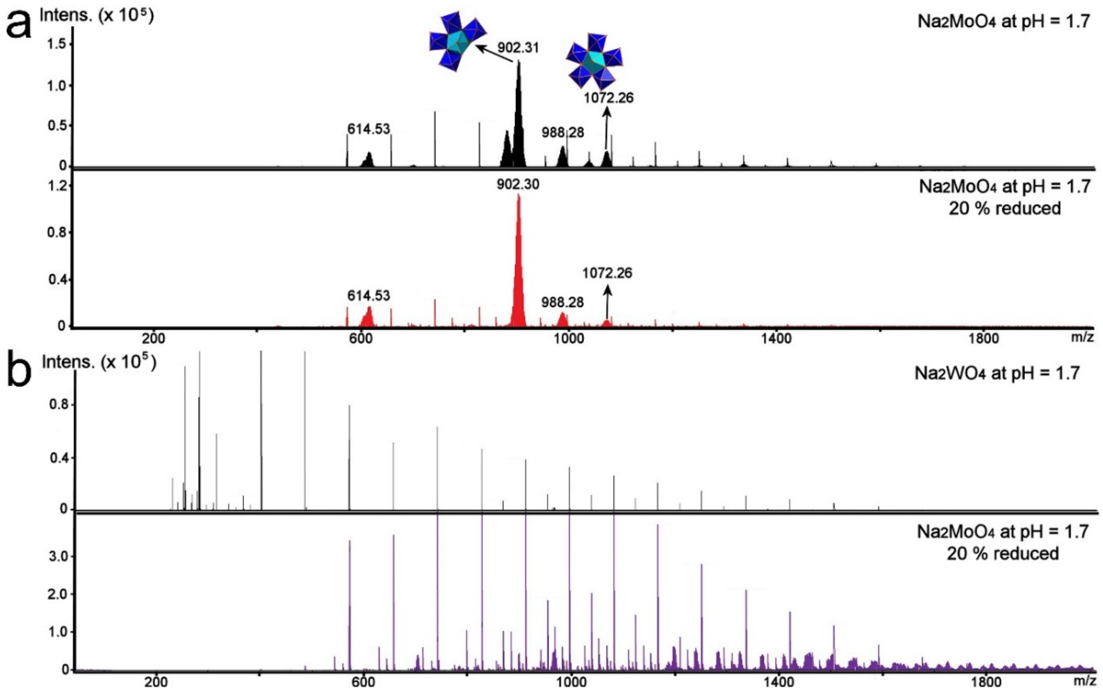

Figure S34. Reaction mixture speciation. a. Representation on the ESI-MS spectrum of sodium molybdate (0.2 M) aqueous solution at pH = 1.7 acidified with HNO_3 (2 M) before and after 20% reduction of the molybdenum content using $Na_2S_2O_4$. The presence of the autocatalytic sets in the molybdenum systems uses effectively the constituents of the building block library to form specific species such as {Mo_{36}} or {Mo_{154}} after reduction. The observed distribution envelopes could be assigned to {Mo_6}, the key building block in the molybdenum chemistry. The distribution envelopes centred at 902.4, 988.4 and 1072.3 m/z could be assigned to $[Mo_5O_{24}H_{16}Na]^-$, $[Mo_6O_{25}H_{13}]^-$ and $[Mo_6O_{26}H_{12}Na_3]^-$ respectively. b. Representation on the ESI-MS spectrum of sodium tungstate (0.2 M) aqueous solution at pH = 1.7 acidified with HNO_3 (2 M) before and after 20% reduction of the tungsten content using $Na_2S_2O_4$. In marked contrast, acidification and reduction of the reaction mixture resulted in a combinatorial explosion of various species and nuclearities due to the numerous energetically comparable possibilities. In this case, the absence of similar autocatalytic set was crucial for the lack of organization of the generated library into specific and well-defined molecular objects.

Electrospray ionization mass spectrometry was performed using a Bruker micrOTOF-Q quadrupole time-of-flight mass spectrometer. Samples were dissolved in methanol introduced at a dry gas temperature of 180 °C. The ion polarity for all MS scans recorded was negative, with the voltage of the capillary tip set at 4500 V, end plate offset at –500 V, funnel 1 RF at 400 Vpp and funnel 2 RF at 400 Vpp, hexapole RF at 200 Vpp, ion energy 5.0 eV, collision energy at 15 eV, collision cell RF at 1200 Vpp, transfer time at 120.0 μs, the pre-pulse storage time at 15.0 μs and analysed using the Bruker Daltonics v4.1 software.

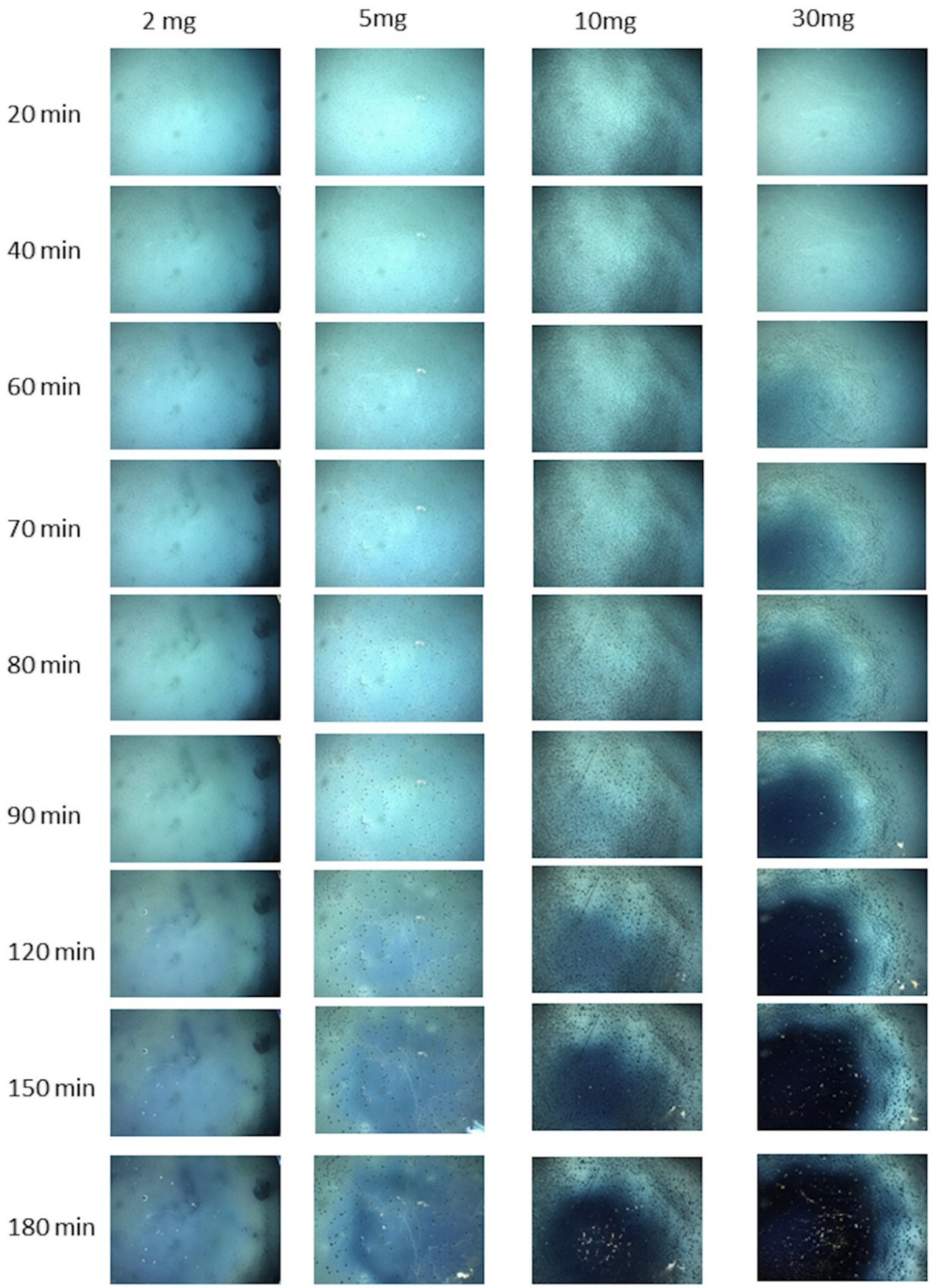

Figure S35. Effect of Keggin concentration of the formation of 1. The following scaled down synthetic procedure was used for the recording purposes of the crystallisation; $7MoO_3.Ce_2O_3$ (200 mg) was dispersed in 8 mL H_2O, then 1.8 mL 1M HClO4, 0.6 mL 0.5 M cysteine in the presence of 2, 5, 10

and 30 mgs of $H_3PMo_{12}O_{40}$. The mixture was heated at 90 °C for 1-2 h and filtered while hot. The hot filtrate was placed immediately in petri dishes (t=0). Observation of the $\{PMo_{12}\}\subset\{Mo_{124}Ce_4\}$ crystallization at: 20 min; 40 min; 60 min; 90 min; 120 min; 150 min and 180 min. The presence even of tiny amounts of Keggin template triggered the formation of the 1 almost immediately (first traces of crystalline material could be observed even at 5-10 min in every case). However, increased concentrations of Keggin template induced the formation and subsequent crystallisation of larger amounts of 1 at the same period of time.

7: Crystallographic data

Data were collected at 150(2) K using a Bruker AXS Apex II [λ(MoKα) = 0.71073 Å] equipped with a graphite monochromator. Suitable single crystals were selected and mounted onto a rubber loop using Fomblin oil. Data collection and reduction were performed using the Apex3 software package and structure solution, and refinement was carried out by SHELXS-2014 and SHELXL-2014. All the Mo atoms (including those disordered), Ce atoms, and most of the O atoms were refined anisotropically. Corrections for incident and diffracted beam absorption effects were applied using empirical absorption corrections. Solvent water molecule sites with partial occupancy were found and included in the structure refinement. The crystallographic formula typically contains many more water molecules in the crystal lattice than those found in the sample after drying. With these types of structures, we are moving outside the realm of small-molecule crystallography, dealing with refinements and problems that lie between small-molecule and protein crystallography. As a result, the refinement statistics are similar to those found for protein structures. However, the final refinement statistics are of publishable quality, and the structural analysis allows us to unambiguously determine the structure of the compound. Final unit cell data and refinement statistics for the compound **1** are collated in Tables S2 and S3. Crystallographic data for compound **1** (CCDC 1916468) can be obtained free of charge from the Cambridge Crystallographic Data Centre, 12, Union Road, Cambridge CB2 1EZ; fax:(+44) 1223–336–033, deposit@ccdc.cam.ac.uk.

Supplementary Table 1. Simulation Parameters for graphs shown in manuscript and SI

Parameter	Figure 3 (b & c)	Figure 4 (a)	SI Figure 26 & 27	SI Figure 28	SI Figure 29, 30, 31	SI Figure 32
**Mo particles**	10^6	10^6	10^6	10^6	10^6	10^6
**Run time**	100	100	100	100	100	5
$\boldsymbol{k_f}$	$3 * 10^{-4}$	$3 * 10^{-4}$	Varying	$3 * 10^{-4}$	$3 * 10^{-4}$	$3 * 10^{-4}$
$\boldsymbol{k_d}$	0.001	0.001	0.001	0.001	0.001	0.001
$\boldsymbol{k_{Mo36}}$	10.0	10.0	0.0	Varying	10.0	10.0
$\boldsymbol{k_{nano}}$	100.0	100.0	0.0	0.0	Varying	100.0
$\boldsymbol{D}$	Varying	Varying	Varying	Varying	Varying	10^{-4}

Table S2. Crystal data and structure refinement details for **1**

Empirical formula	$C_{48}H_{632}Ce_4KMo_{136}N_{16}O_{704}PS_{16}$
Formula weight	26893.01 g mol^{-1}
Temperature	150(2) K
Wavelength	71.073 pm
Crystal system	Monoclinic
Space group	*C* 2/m
Unit cell dimensions	a = 5015.3(4) pm, α = 90°
	b = 4179.9(4) pm, β = 97.056(6)°
	c = 3673.8(3) pm, γ = 90°
Volume	76.43(1) nm^3
Z	4
Density (calculated)	2.337 Mg/m^3
Absorption coefficient	2.546 mm^{-1}
F(000)	51592
Crystal size	0.100 x 0.050 x 0.050 mm^3
Theta range for data collection	0.636 to 25.886°.
Index ranges	-61<=h<=60, 0<=k<=51, 0<=l<=44
Reflections collected	44392
Independent reflections	44392 [R(int) = 0.0561]
Max. and min. transmission	0.562 and 0.446
Refinement method	Full-matrix least-squares on F^2
Data / restraints / parameters	44392 / 1576 / 3633
Goodness-of-fit[c]	1.131
Final R indices [I>2sigma(I)]	R1[a] = 0.0995, wR2[b] = 0.2440

[a] $R1 = \Sigma||F_o| - |F_c||/\Sigma|F_o|$. [b] $wR2 = \{\Sigma[w(F_o^2 - F_c^2)^2]/\Sigma[w(F_o^2)^2]\}^{1/2}$, where $w = 1/[\sigma^2(F_o^2) + (aP)^2 + bP]$, $P = (F_o^2 + 2F_c^2)/3$. [c] $GoF = \{\Sigma[w(F_o^2 - F_c^2)^2]/(n - p)\}^{1/2}$, where n = number of reflections and p is the total number of parameters refined.

Table S3. Selected Bond Distances [pm] (Å) for **1**

Bond	Length	Bond	Length	Bond	Length	Bond	Length
Mo(1)-O(10)	173(2)	Mo(6)-O(46)	166(1)	Mo(12)-O(210)	168(1)	Mo(71)-O(280)	235(3)
Mo(1)-O(8)	178(2)	Mo(6)-O(40)	192(1)	Mo(12)-O(206)	184(1)	Mo(72)-O(173)	175(3)
Mo(1)-O(5)	192(1)	Mo(6)-O(42)	193(1)	Mo(12)-O(263)	192(1)	Mo(72)-O(276)	184(3)
Mo(2)-O(6)	165(1)	Mo(7)-O(50)	169(1)	Mo(13)-O(83)	169(2)	Mo(72)-O(276)[#]	184(3)
Mo(2)-O(1)	184(1)	Mo(7)-O(48)	172(1)	Mo(13)-O(205)	189(1)	Mo(72)-O(277)	201(2)
Mo(2)-O(11)	190(1)	Mo(7)-O(43)	186(1)	Mo(13)-O(104)	198(1)	Mo(72)-O(280)	251(3)
Mo(2)-O(9)	203(1)	Mo(8)-O(76)	168(1)	Mo(14)-O(195)	169(1)	Mo(73)-O(291)	173(2)
Mo(3)-O(20)	164(1)	Mo(8)-O(87)	188(1)	Mo(14)-O(263)	186(1)	Mo(73)-O(277)	182(2)
Mo(3)-O(11)	185(1)	Mo(8)-O(74)	197(1)	Mo(14)-O(194)	188(1)	Mo(73)-O(292)	186(4)
Mo(3)-O(21)	189(1)	Mo(9)-O(80)	166(1)	Mo(69)-O(283)	241(2)	Mo(73)-O(215)	190(3)
Mo(3)-O(41)	201(1)	Mo(9)-O(79)	186(1)	Mo(70)-O(282)	172(2)	Mo(73)-O(299)	190(1)
Mo(4)-O(25)	165(1)	Mo(9)-O(86)	191(1)	Mo(70)-O(287)	174(3)	Mo(73)-O(289)	236(3)
Mo(4)-O(23)	183(1)	Mo(10)-O(92)	168(1)	Mo(70)-O(283)	246(1)	Mo(74)-O(293)	162(3)
Mo(4)-O(21)	186(1)	Mo(10)-O(91)	177(1)	Mo(71)-O(275)	176(2)	Mo(74)-O(288)	190.8(8)
Mo(4)-O(26)	201(1)	Mo(10)-O(261)	193(1)	Mo(71)-O(279)	190(4)	P(1)-O(289)	154(2)
Mo(5)-O(33)	169(1)	Mo(11)-O(211)	169(1)	Mo(71)-O(276)	190(2)	P(1)-O(289)[#1]	154(2)
Mo(5)-O(37)	195(1)	Mo(11)-O(89)	194(1)	Mo(71)-O(278)	193(2)	P(1)-O(280)	157(4)
Mo(5)-O(26)	196(1)	Mo(11)-O(85)	199(1)	Mo(71)-O(274)	196(2)	P(1)-O(283)	157(3)
